# Personal, professional, and psychological impact of the COVID-19 pandemic on hospital workers: A cross-sectional survey

**DOI:** 10.1371/journal.pone.0263438

**Published:** 2022-02-15

**Authors:** Kimia Honarmand, Christopher J. Yarnell, Carol Young-Ritchie, Robert Maunder, Fran Priestap, Mohamed Abdalla, Ian M. Ball, John Basmaji, Chaim M. Bell, Lianne Jeffs, Sumesh Shah, Jennifer Chen, Danielle LeBlanc, Jessica Kayitesi, Catherine Eta-Ndu, Sangeeta Mehta

**Affiliations:** 1 Department of Medicine- Division of Critical Care, Western University, London, Ontario, Canada; 2 Department of Medicine, Sinai Health System, Toronto, Ontario, Canada; 3 Interdepartmental Division of Critical Care Medicine, University of Toronto, Toronto, Ontario, Canada; 4 London Health Sciences Centre, London, Ontario, Canada; 5 Department of Psychiatry, Sinai Health System and University of Toronto, Toronto, Ontario, Canada; 6 Department of Surgery- Trauma Program, London Health Sciences Centre, London, Ontario, Canada; 7 Department of Medicine, Tillsonburg District Memorial Hospital, Tillsonburg, Ontario, Canada; 8 Department of Epidemiology and Biostatistics, Western University, London, Ontario, Canada; 9 Departments of Medicine and Health Policy Management and Evaluation, University of Toronto, Toronto, Ontario, Canada; 10 Lunenfeld-Tanenbaum Research Institute, Sinai Health System, Toronto, Ontario, Canada; 11 Bloomberg Faculty of Nursing and Institute of Health Policy, Management, and Evaluation, University of Toronto, Toronto, Ontario, Canada; 12 Department of Medical Biophysics, Western University, London, Ontario, Canada; 13 Department of Nursing, Sinai Health System, Toronto, Ontario, Canada; St John’s University, UNITED KINGDOM

## Abstract

**Objectives:**

We aimed to evaluate the personal, professional, and psychological impact of the COVID-19 pandemic on hospital workers and their perceptions about mitigating strategies.

**Design:**

Cross-sectional web-based survey consisting of (1) a survey of the personal and professional impact of the COVID-19 pandemic and potential mitigation strategies, and (2) two validated psychological instruments (Kessler Psychological Distress Scale [K10] and Impact of Events Scale Revised [IES-R]). Regression analyses were conducted to identify the predictors of workplace stress, psychological distress, and post-traumatic stress.

**Setting and participants:**

Hospital workers employed at 4 teaching and 8 non-teaching hospitals in Ontario, Canada during the COVID-19 pandemic.

**Results:**

Among 1875 respondents (84% female, 49% frontline workers), 72% feared falling ill, 64% felt their job placed them at great risk of COVID-19 exposure, and 48% felt little control over the risk of infection. Respondents perceived that others avoided them (61%), reported increased workplace stress (80%), workload (66%) and responsibilities (59%), and 44% considered leaving their job. The psychological questionnaires revealed that 25% had at least some psychological distress on the K10, 50% had IES-R scores suggesting clinical concern for post-traumatic stress, and 38% fulfilled criteria for at least one psychological diagnosis. Female gender and feeling at increased risk due to PPE predicted all adverse psychological outcomes. Respondents favoured clear hospital communication (59%), knowing their voice is heard (55%), expressions of appreciation from leadership (55%), having COVID-19 protocols (52%), and food and beverages provided by the hospital (50%).

**Conclusions:**

Hospital work during the COVID-19 pandemic has had important personal, professional, and psychological impacts. Respondents identified opportunities to better address information, training, and support needs.

## Introduction

The COVID-19 pandemic has compelled frontline healthcare workers (HCWs) to risk their personal safety in providing patient care. Such challenging circumstances have adverse effects on frontline HCWs. In the aftermath of the 2003 severe acute respiratory syndrome (SARS) pandemic, HCWs experienced symptoms of depression, anxiety, and chronic stress [1–5] that persisted for years [3].

The COVID-19 pandemic presents unique challenges to HCWs [6–10]. Concerns about personal and family safety are compounded by worries about a surge of patients, depletion of hospital resources (e.g., ventilators, personal protective equipment [PPE]), and rapidly-changing direction from various levels of authority (e.g., PPE recommendations). In contrast to prior public health emergencies (i.e., SARS), our current digital age provides a wealth of on-demand, unverified information that predisposes HCWs to cognitive fatigue. Lastly, physical distancing, while an effective strategy to control the spread of this disease, may lead to personal isolation and a loss of support systems that are vital for HCWs’ psychological wellbeing.

Studies conducted in the aftermath of the SARS outbreak reported that being in the nursing profession [2], being a frontline worker [4, 5], female gender [5], relationship status [2], and living with a child or children [4] were predictive of increased psychological burden among HCWs. Similarly, emerging evidence from studies during the COVID-19 pandemic suggest that younger age [9, 11], working in a community hospital [7, 9, 10, 12], and knowing someone with COVID-19 [6] are additional predictors of increased psychological burden among HCWs.

HCWs are fundamental to the functioning of the increasingly stressed health care system and are the most limited resource in many jurisdictions [13, 14]. There is an urgent need to determine the scope of the pandemic’s impact on the healthcare workforce and identify and implement public health mitigation strategies [15]. The purpose of this study is to characterize the personal, professional, and psychological impact of the COVID-19 pandemic among teaching and non-teaching hospital workers.

## Methods

We conducted a web-based survey of workers, including nurses, physicians, other healthcare professionals, as well as administrative, research, and other hospital staff, at 4 teaching and 8 non-teaching hospitals across 2 regions in Ontario (Toronto and Southwest Ontario [SWO]). The checklist for reporting results of internet e-surveys (CHERRIES) was used for a more complete description of the survey methodology [S1 Appendix] [16]. The study was approved by the Sinai Health (20-0089-E) and Western University (#115850) Research Ethics Boards; and consent was implied by survey completion.

### Survey instruments

We adapted a survey previously used during the SARS pandemic [2, 5, 8]. Using formal survey development methodology [17], the research team iteratively refined the existing instrument and engaged representatives from stakeholder groups (nurses, physicians (including a psychiatrist), health disciplines professionals, and researchers with pandemic expertise) for pre-testing to ensure that questions addressed concerns specific to hospital staff, and were likely to yield information pertinent to the study objective. The survey was available only in English. Responses consisted primarily of attitude statements scored on a six-point Likert scale ranging from (1) Strongly Disagree to (6) Strongly Agree, with an option for free-text responses to several survey items [S2 Appendix].

For respondents in the Southwest Ontario cohort (10 hospitals), we also administered two validated psychological instruments: the Kessler Psychological Distress Scale (K10) [18] and the Impact of Events Scale Revised (IES-R) [19]. Both have been widely validated [K10 [20, 21]; IES-R [22, 23]] and used to assess the psychological impact of the COVID-19 [6, 7, 24, 25] and the SARS pandemics [2, 4, 5, 8].

### Sample size calculation

We derived a minimum sample size estimate of 346 using standard survey sample size calculation that incorporates population size, confidence level of 95% and confidence interval of 5%. We aimed to collect a minimum of 1500 complete responses to enable subgroup analyses.

### Settings & survey administration

The survey was distributed across 12 hospitals in Ontario over several weeks in July and September 2020, using one of two secure, web-based platforms: NoviSurvey for Toronto sites and REDCap^®^ for Southwest Ontario sites. Participants were invited via email by local hospital leadership to complete the survey, with 2 to 3 reminders over several weeks. In addition, the survey invitation and link were posted on the hospital COVID-19 research page for Toronto sites. Survey announcements and email invitations are included in S3 Appendix.

### Data analysis

We summarized responses using descriptive statistics: proportions, means and standard deviation (SD), and medians and interquartile ranges (IQR), as appropriate. For Likert-scale questions, we summarized responses according to the proportion of respondents that agreed (either strongly agree, agree, or unsure but probably agree) with each item.

We reported the K10 total score, and depression and anxiety subscores using descriptive statistics [S1 Table]. We performed independent-samples Kruskal-Wallis Test to compare nursing professionals, physicians, and other hospital staff on the K10 psychological distress, depression subscore, anxiety subscore, and IES-R score.

After conducting regression diagnostics (assumption testing), we performed ordinal regression analysis to identify predictors of increased workplace stress (strongly agree, agree, or unsure but probably agree with the statement: “*I have felt more stressed at work*”) and linear regression analysis to identify predictors of K10 total score, depression subscore, and anxiety subscore, and IES-R score. For all outcomes, we selected 11 predictor variables that have shown an association with psychological symptom burden during the SARS outbreak [4, 5, 8] and the COVID-19 pandemic [9, 10]: five predictors related to professional activities (working in a teaching vs. non-teaching hospital, nursing vs. other professions, being a frontline worker, years of healthcare experience, and feeling at increased personal risk due to PPE shortage or inadequate PPE training), and six related to demographic characteristics (age group, gender, high-risk health condition, relationship status, living with one or more children, and knowing someone who contracted COVID-19) [S2 Table]. All variables were entered into the initial regression model and purposefully selected according to the approach described by Bursac and colleagues (2008) [26]. We retained those variables that yielded an association with the outcome variables with a p-value < 0.1 in the final regression model [S2 Table]. We performed all statistical analyses using Statistical Package for Social Sciences Version 25.0 (IBM Corp, 2017; Armonk, NY, USA).

Two investigators (KH and DL) performed qualitative analysis of all open-ended responses using thematic content analysis methodology [27]. First, they coded each open-ended response independently and in duplicate. Then, in a series of coding meetings, they generated themes and subthemes related to the personal and professional impact of the COVID-19 pandemic on HCWs.

## Results

Between July and September 2020, 1875 individuals (84% female) completed the survey and were included in the analysis. The sample consisted of nurses (n = 623, 33%), physicians (n = 168, 9%), other health discipline professionals (n = 441, 24%), among other hospital workers (n = 643, 34%). Among these, 923 (49%) were frontline workers (reported caring for patients with suspected or confirmed COVID-19). Table 1 presents respondents’ professional and demographic characteristics.

**Table 1:** Respondents’ professional and demographic characteristics.

Characteristic	All respondents	Respondents to K10 and IES-R questionnaires
	N = 1875	
		N = 962
**Profession**		
Nursing^a^	623 (33.2)	403 (41.9)
Physician	168 (9.0)	81 (8.4)
Other health discipline professionals^b^	441 (23.5)	249 (25.9)
Administrative	260 (13.9)	103 (10.7)
Research	145 (7.7)	5 (0.5)
Management	74 (3.9)	31 (3.2)
Other hospital staff^c^	164 (8.8)	90 (9.4)
**Frontline worker** ^d^		
Yes	923 (49.2)	568 (59.0)
No	952 (50.8)	394 (41.0)
**Years of healthcare experience**		
≤ 5 years	500 (26.7)	242 (25.2)
6–10 years	377 (20.1)	214 (22.2)
11–20 years	505 (26.9)	240 (24.9)
> 20 years	488 (26.0)	266 (27.7)
No response	5 (0.3)	0 (0)
**Age group**		
< 30 years	399 (21.3)	226 (23.5)
31–40 years	506 (27.0)	255 (26.5)
41–50 years	441 (23.5)	214 (22.2)
51–60 years	424 (22.6)	235 (24.4)
≧61 years	97 (5.1)	30 (3.1)
No response	8 (0.4)	2 (0.2)
**Gender**		
Female	1569 (83.7)	844 (87.7)
Male	266 (14.2)	110 (11.4)
Non-binary	4 (0.2)	1 (0.1)
Prefer not to respond	36 (1.9)	7 (0.7)
**Ethnicity** ^e^		
White	1438	864
Black	38	5
Middle Eastern	22	11
Indigenous	13	11
Central and East Asian	115	20
South Asian	90	15
Southeast Asian	37	7
West Asian	10	3
Other	41	16
Prefer not to respond or no response	86	27
**Marital Status**		
Married or common-law	1249 (66.6)	673 (70.0)
Single	443 (23.6)	199 (20.7)
Widowed	15 (0.8)	6 (0.6)
Divorced or separated	114 (6.1)	59 (6.1)
No response	54 (3.1)	25 (2.6)
**High-risk health condition** ^f^		
Yes	431 (23.0)	218 (22.7)
No	1439 (76.7)	740 (76.9)
No response	5 (0.3)	4 (0.4)
**Living arrangement** ^e^		
Alone	229 (12.2)	109 (11.3)
Partner or spouse	1296 (69.1)	699 (72.7)
Child or children	826 (44.1)	431 (44.8)
Extended family	201 (10.7)	79 (8.2)
Roommates	63 (3.4)	31 (3.2)
Caregiver or nanny	5 (0.3)	2 (0.2)
Someone who is 65 years or older	82 (4.4)	26 (2.7)
Someone who is immunocompromised	107 (5.7)	53 (5.5)
**Single parent**		
Yes	155 (14.9)	73 (7.6)
No	888 (85.1)	490 (50.9)

Data are presented as N (%). Respondents from Southwest Ontario (11 hospitals) completed the K10 and IES-R questionnaires.

^a^ Includes registered nurses, registered practical nurses, & nurse practitioners.

^b^ Includes registered healthcare professionals (e.g., psychologists, registered midwives, respiratory therapists, physiotherapists, occupational therapists, registered dieticians, laboratory technicians, etc.).

^c^ Includes all other hospital staff not included in other categories.

^d^ Reported caring for suspected and/ or confirmed covid-19 patients.

^e^ Total responses are greater than total sample size because some respondents selected more than one response choice.

^f^ Reported having a health condition or taking medications that places them at higher risk of poor outcomes if they were to contract COVID-19.

### Perception of risk

**Fig 1:**
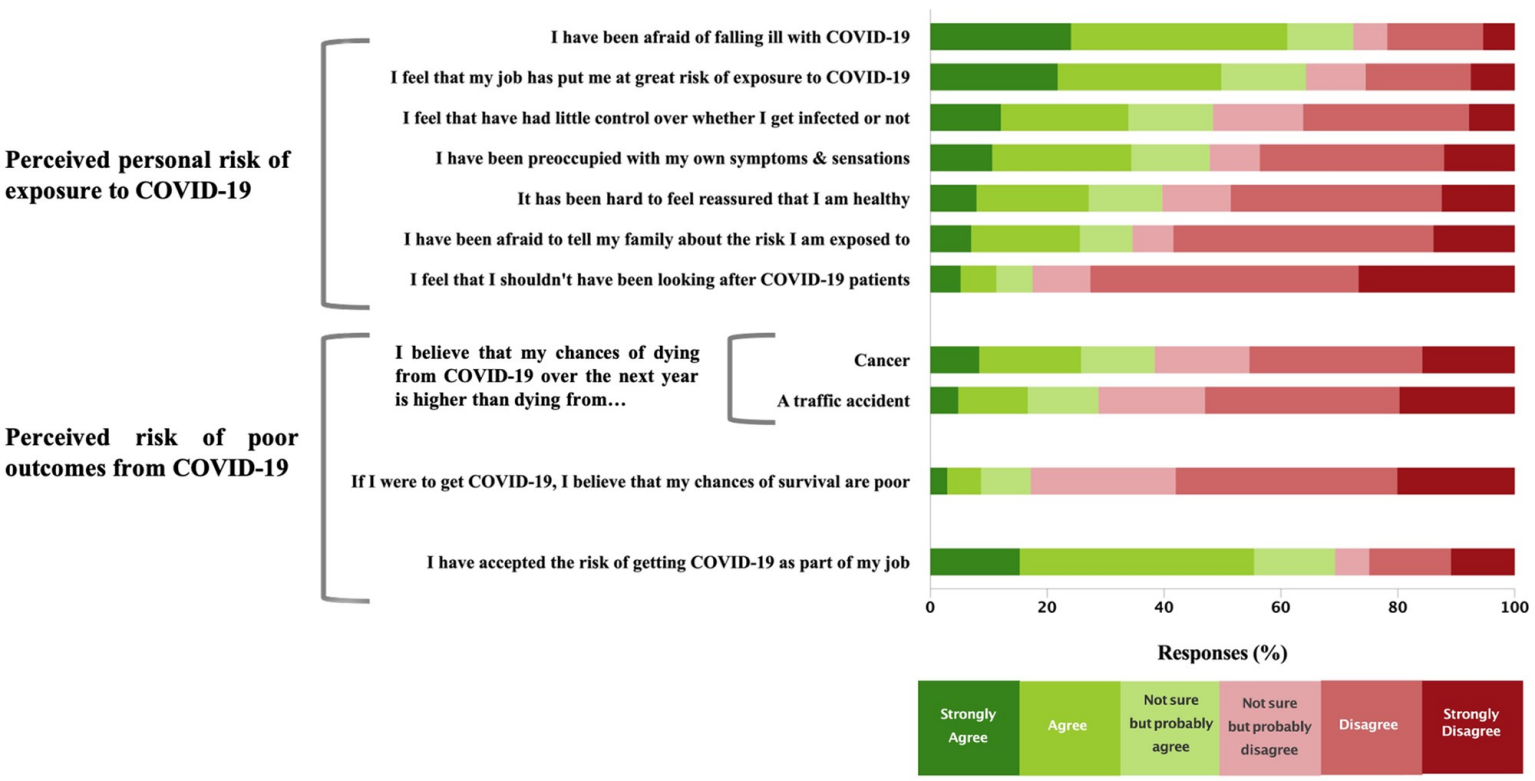
Perceived risks of personal exposure & poor outcomes among hospital workers during the COVID-19 pandemic.

Fig 1 shows respondents’ perceptions of exposure risk and associated outcomes from COVID-19. Overall, 72% reported being afraid of falling ill and 64% felt that their job placed them ‘at a great risk of exposure’ to COVID-19. Almost half (48%) reported feeling that they had little control over whether they got infected, 48% reported being preoccupied with their own symptoms, and 40% found it hard to feel reassured of their health. More than one-third of respondents were afraid to tell their family about their professional exposure risk.

A notable proportion felt that their chance of dying from COVID-19 in the next year was higher than dying from cancer (38%) or a traffic accident (29%), and 17% felt their chances of survival were poor if they contracted COVID-19. Despite these fears, 69% accepted the risk of contracting COVID-19 as part of their job.

Respondents believed they were at high risk of contracting COVID-19 from fomites (992/1755, 57%), COVID-19 patients (873/1608, 54%), colleagues (908/1704, 53%), shortage of PPE (851/1731, 49%), the air that they breathed (637/1725, 37%), and inadequate PPE training (374/1719, 22%).

Respondents expressed concerns about transmitting COVID-19 to people close to them outside of work (876/1707, 51%), particularly family members (1474/1703, 87%), friends (1087/1648, 66%), colleagues (1145/1692, 68%), and patients (760/1341, 57%).

### Personal impact of the COVID-19 pandemic: Exposures and experiences

Few respondents reported knowing someone who contracted COVID-19 within their immediate family (72/1875, 4%), friends (110/1875, 5.9%), community (231/1875, 12%), acquaintances (261/1875, 14%), and colleagues (409/1875, 22%); only 8 (0.4%) reported that someone in their home had confirmed COVID-19. One in 4 respondents (448/1875, 24%) had been separated from their family because of COVID-19. School closures meant that 28% (252/901) respondents had stayed home to provide childcare.

Regarding perceived stigmatization due to their profession, 61% (981/1612) felt that people avoided them, 39% (628/1594) felt that people avoided their family members, and 35% (557/1615) have avoided telling people about the nature of their job.

### Professional impact of the COVID-19 pandemic

#### Workplace exposures, workload, and coping

Overall, 56% respondents (1043/1875) reported that COVID-19 patients had been treated in their clinical area. Respondents had personally attended to patients with confirmed (653/1875, 35%) and suspected (899/1875, 48%) COVID-19, with 60% (1116/1875) reporting direct daily contact with suspected or confirmed COVID-19 patients.

**Fig 2:**
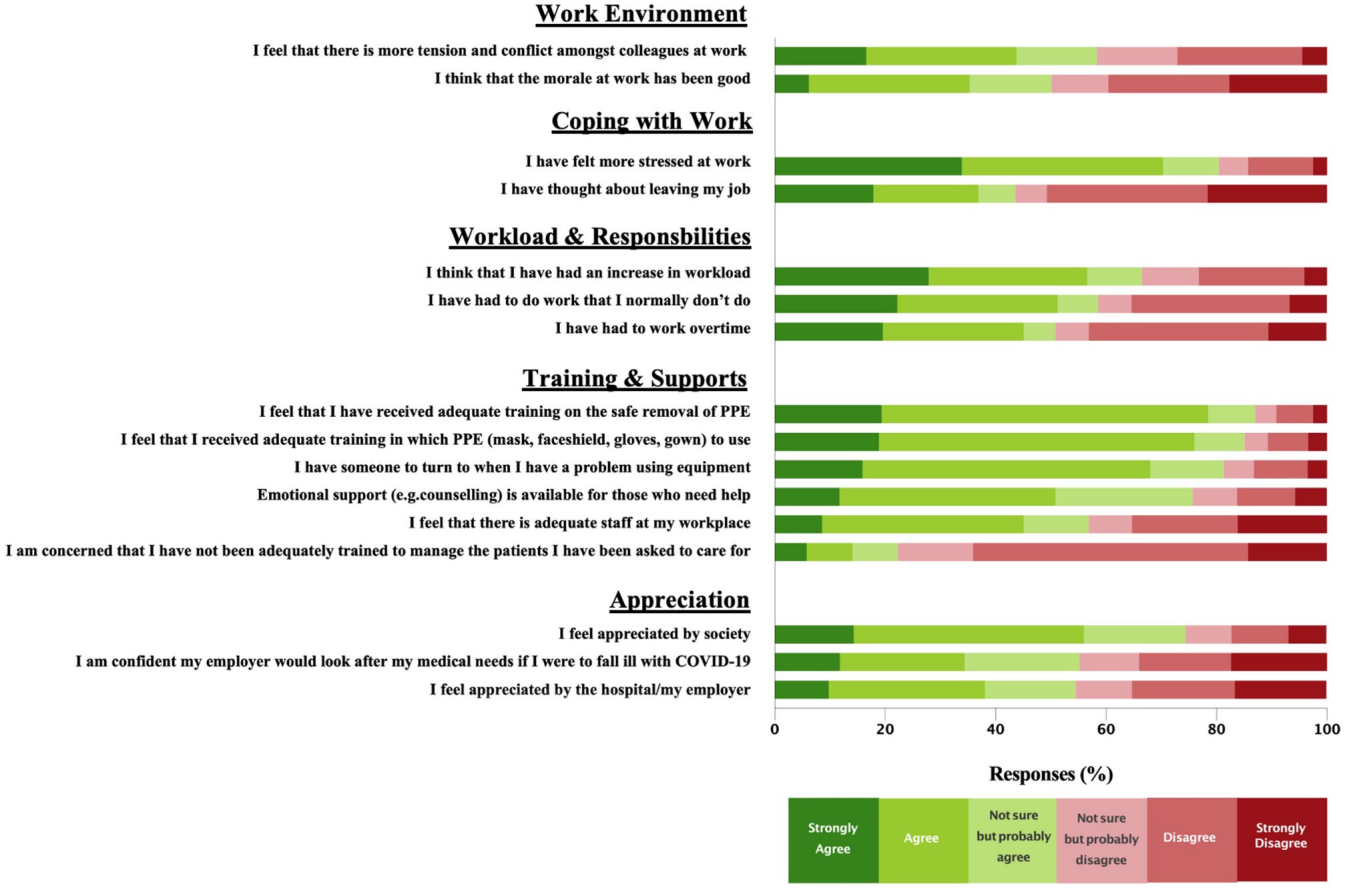
The professional impact of the COVID-19 pandemic on hospital workers.

Respondents endorsed increased workplace stress (80%), workload (1085/1633, 66%), responsibilities (954/1627, 59%), and working overtime (795/1565, 51%) during the pandemic; and 44% had contemplated leaving their job (Fig 2). Although 58% respondents felt more tension and conflict amongst colleagues, 44% felt that morale had been good.

#### Training, supports, and appreciation

Most respondents felt well-supported at work in the form of adequate staffing (929/1632, 57%), having a resource for PPE challenges (1221/ 1500, 81.4%), and availability of counseling (1201/1586, 76%). Most respondents endorsed adequate training in PPE use (1311/1543, 85%) and removal (1344/1546, 87%). Among Southwest Ontario respondents, 22% (199/892) reported concern about inadequate training to care for COVID-19 patients (Fig 2).

More than half (949/1720, 55%) expressed confidence that their employer would look after their medical needs if they were to fall ill with COVID-19, and a similar proportion (899/1648, 55%) felt appreciated by their hospital. In comparison, 75% (1209/1624) reported feeling appreciated by society in general.

### Psychological state among hospital workers during the COVID-19 pandemic

Table 1 summarizes the demographic characteristics of the 962 respondents in the Southwest Ontario survey who completed the K10 and IES-R scales. Overall, 56% (480/861) had more than mild symptoms of psychological distress or post-traumatic stress, and 38% (317/839) had concern for at least one psychological diagnosis based on the two instruments.

#### Psychological distress and symptoms of depression and anxiety (Fig 3; S3 Table)

**Fig 3:**
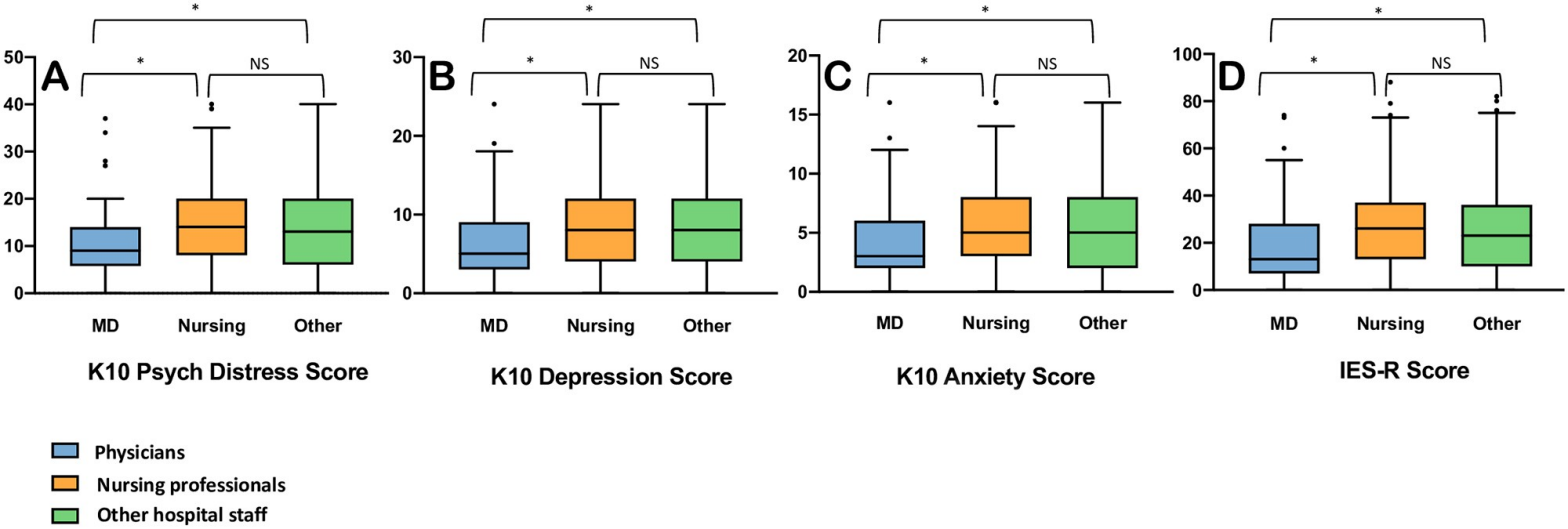
Box plots with Tukey whiskers demonstrating scores on psychological questionnaires among hospital workers during the COVID-19 pandemic by professional role. [* indicates a significant difference was found in the pairwise comparisons, p < 0.005; Not significant **(NS)**, p > 0.05]. **K10**: Kessler Psychological Distress Scale; **IES-R**: Impact of Events Scale Revised. **A**. K10 psychological distress—Physicians vs. Nursing: H = -121.24, p < 0.001; Physicians vs. Other: H = -103.43, p = 0.002; Other vs. Nursing: H = 17.81, p = 0.33. **B**. K10 Depression subscore—Physicians vs. Nursing: H = -116.20, p < 0.001; Physicians vs. Other: H = -104.50, p = 0.001; Other vs. Nursing: 11.702, p = 0.64. **C**: K10 Anxiety subscore—Physicians vs. Nursing: H = -117.69, p < 0.001; Physicians vs. Other: H = -96.49, p = 0.003; Other vs. Nursing: H = 21.20, p = 0.25. **D**: IES-R total score—Physicians vs. Nursing: H = -134.66, p < 0.001; Physicians vs. Other: H = -109.69, p < 0.001; Other vs. Nursing: H = 24.97, p = 0.16.

Among 923 respondents who completed all K10 items, 233 (25%) had at least some psychological distress: 126 (14%) had mild, 63 (7%) had moderate, and 44 (5%) had severe psychological distress. Compared to physicians, nursing professionals and other hospital workers had higher K10 total scores, as well as depression and anxiety subscores (Fig 3; S3 Table). For the depression subscore, respondents most frequently rated two fatigue-related symptoms as ‘most of the time’ or ‘all of the time’: feeling tired for no good reason (375/954, 39%) and feeling that everything is an effort (215/948, 23%). Among depressive symptoms suggesting negative affect, respondents reported feeling depressed (153/954, 16%), hopeless (107/954, 11%), and worthless (107/948, 11%), and 9% (88 of 952) reported feeling so depressed that nothing could cheer them up. Regarding anxiety symptoms, respondents most frequently rated two items related to nervousness as ‘most of the time’ or ‘all of the time’: feeling nervous (262/952, 28%), and feeling so nervous that nothing could calm them down (70/953, 8%). Respondents also endorsed the two agitation-related items: feeling restless or fidgety (184/955, 19%) and feeling so restless that they could not keep still (97/953, 10%).

#### Post-traumatic stress symptoms (Fig 3; S3 Table)

Half (423/849) of the respondents had IES-R scores suggestive of concern for clinical PTSD, with 16% (134/849) having partial PTSD (at least some symptoms), and 34% (289/849) meeting criteria for probable PTSD. Of all respondents, 24% (206/849) scored 37 or higher, which has been associated with suppressed immune function for up to 10 years following the inciting event [28]. Compared to physicians, nursing professionals and other hospital workers had higher IES-R scores (Fig 3; S3 Table). Respondents most frequently endorsed difficulty staying asleep (288/957, 30%), falling asleep (256/952, 27%), and feeling irritable and angry (284/956, 30%), ‘quite a bit’ or ‘extremely’.

### Predictors of workplace stress and psychological symptoms on IES-R and K10

There were no violations of the assumption testing on ordinal and regression analyses. On multivariable ordinal regression, predictors of increased workplace stress (responding strongly agree or agree to the question: “I have felt more stressed at work”) included female gender, having a high-risk health condition, younger age, personally knowing someone who contracted COVID-19, working in a non-teaching (relative to a teaching) hospital, and feeling increased personal risk due to PPE shortage or inadequate training (Table 2).

**Table 2:** Multivariable analysis of the predictors of increased workplace stress among hospital workers during the COVID-19 pandemic.

Predictors	Increased workplace stress
	Coefficient
	(95% CI)
**Age *[reference group*: *> 50 years of age]***	
< = 30 years	0.256
	(-0.018 to 0.530)
31–40 years	0.445
	(0.192 to 0.698)
41–50 years	0.348
	(0.087 to 0.608)
**Female gender**	0.517
	(0.258 to 0.776)
**High risk health status** ^a^	0.437
	(0.208 to 0.666)
**Not married or common-law relationship**	-
**Living with a child or children**	-
**Personally know someone who had COVID-19**	0.316
	(0.124 to 0.508)
**Non-teaching hospital**	0.228
**[vs. teaching]**	
	(0.021 to 0.434)
**Nursing profession**	-
**[vs. other]**	
**Frontline worker** ^b^	-
**Years of healthcare experience *[reference group*: *> 20 years of healthcare experience]***	
< = 5 years	-
6–10 years	-
11–20 years	-
**Feeling at increased risk due to PPE shortage or inadequate PPE training**	0.900
	(0.707 to 1.093)

CI: confidence interval; PPE: Personal protective equipment

Dash (-) indicates no statistically significant association found.

^a^ Reported having a health condition or taking medications that places them at higher risk of poor outcomes if they were to contract COVID-19.

^b^ Reported caring for suspected and/ or confirmed COVID-19 patients.

On multivariable linear regression, predictors of higher psychological distress (K10 total score) included younger age, female gender, having a high-risk health condition, personally knowing someone who contracted COVID-19, and feeling increased personal risk due to PPE shortage or inadequate training. Table 3 shows the final multivariable regression models for K10 psychological distress, as well as depression and anxiety subscores. Predictors of higher symptoms of post-traumatic stress on the IES-R included female gender, having a high-risk health condition, and feeling increased personal risk due to PPE shortage or inadequate training.

**Table 3:** Multivariable analysis of the predictors of psychological outcomes among hospital workers during the COVID-19 pandemic.

Predictors	K10	K10	K10	IES-R
	Psychological Distress	Depressive Symptoms	Anxiety Symptoms	Post-traumatic stress
	Coefficient	Coefficient	Coefficient	Coefficient
	(95% CI)	(95% CI)	(95% CI)	(95% CI)
**Age *[reference group*: *> 50 years of age]***				
< = 30 years	2.93	1.13	1.59	-
	(1.36 to 4.51)	(0.15 to 2.11)	(0.94 to 2.25)	
31–40 years	2.38	1.25	1.02	-
	(0.86 to 3.89)	(0.30 to 2.20)	(0.39 to 1.65)	
41–50 years	2.66	1.33	1.24	-
	(1.08 to 4.25)	(0.33 to 2.32)	(0.58 to 1.90)	
**Female gender**	2.86	1.57	1.27	8.61
	(1.14 to 4.58)	(0.48 to 2.66)	(0.55 to 2.00)	(5.00 to 12.26)
**High risk health status** ^a^	1.45	-	0.89	3.94
	(0.11 to 2.80)		(0.33 to 1.45)	(1.12 to 6.76)
**Not married or common-law relationship**	-	-	-	-
**Living with a child or children**	-	-	-	-
**Personally know someone who had COVID-19**	1.40	0.99	0.43	-
	(0.26 to 2.53)	(0.27 to 1.70)	(-0.04 to 0.91)	
**Non-teaching hospital**	-	-	-0.41	-
**[vs. teaching]**			(-0.87 to 0.06)	
**Nursing profession**	-	-	-	-
**[vs. other]**				
**Frontline worker** ^b^	-	-	-	-
**Years of healthcare experience *[reference group*: *> 20 years of healthcare experience]***				
< = 5 years	-	-	-	-
6–10 years	-	-	-	-
11–20 years	-	-	-	-
**Feeling at increased risk due to PPE shortage or inadequate PPE training**	3.76	2.26	1.42	9.14
	(2.63 to 4.90)	(1.55 to 2.98)	(0.95 to 1.89)	(6.81 to 11.47)

CI: confidence interval; PPE: Personal protective equipment

Dash (-) indicates no statistically significant association found.

^a^ Reported having a health condition or taking medications that places them at higher risk of poor outcomes if they were to contract COVID-19.

^b^ Reported caring for suspected and/ or confirmed COVID-19 patients.

### Perceptions about protective measures

When asked about specific measures to protect themselves from exposure to COVID-19, the vast majority of respondents endorsed ‘adhering to protocols and recommended measures’ (1599/1621, 99%), avoiding crowded places (1567/1618, 97%), cleaning their environment such as car or home (1378/1614, 85%), and avoiding potentially-exposed colleagues (1132/1523, 74%).

When asked about protective measures implemented at their workplace, most respondents felt they were generally effective (1448/1618, 90%), and that protocols were clear (1007/1613, 62%) and implemented quickly (1046/1608, 65%). Most respondents were satisfied with institutional explanations of their necessity and importance (1397/1618, 86%), reported little difficulty in personally adhering to recommended measures (1366/1602, 85%), and perceived good adherence among other staff (1306/1610, 81%). In contrast, 74% (1203/1612) endorsed that the information and directives provided by the hospital changed too rapidly to keep up, and 70% (1089/1548) felt that frontline HCWs deserved a higher level of personal protection than provided.

Respondents most frequently agreed that the following measures were useful in protecting them from contracting COVID-19: isolation of COVID-19 patients (1588/1613, 99%), enforcing work-from-home for non-essential staff (1482/1599, 93%), personalized mask-fit- testing (1305/1519, 86%), screening of patients and visitors for symptoms (1372/1628, 84%), information provided by their hospital (1333/1637, 81%), daily screening of staff for symptoms (694/1019, 68%), and availability of the occupational health service (1079/1611, 67%). Although 96% of respondents (1568/1634) agreed that limiting the number of hospital visitors was an effective means to protect them from contracting COVID-19, 80% (773/962 SWO respondents) endorsed concerns about the negative impact of visitor restrictions on patients.

### Personal coping strategies

**Fig 4:**
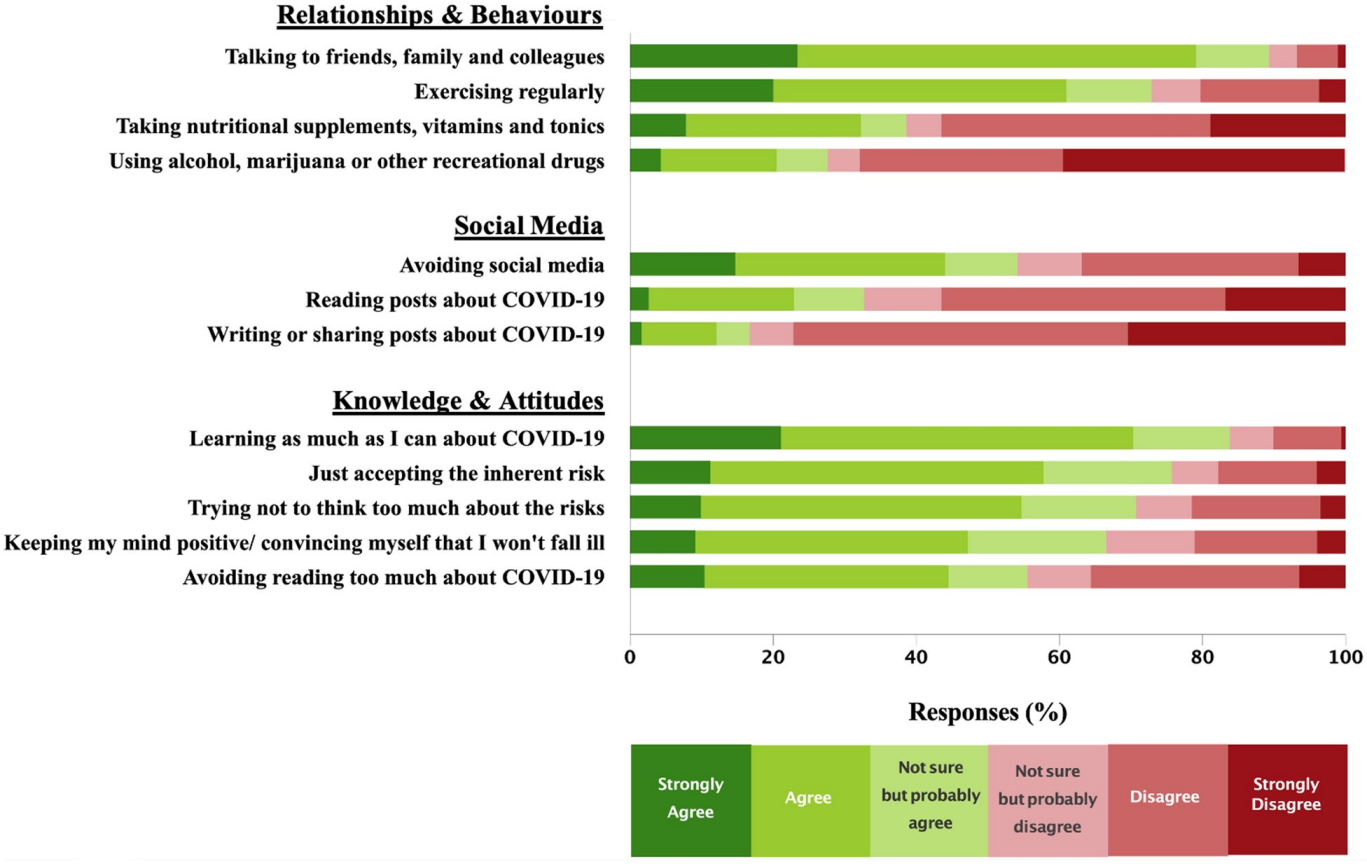
Strategies used by hospital workers to cope with the stresses of the COVID-19 pandemic.

Fig 4 shows personal strategies used by respondents to cope with the pandemic. Most respondents endorsed ‘talking to family and colleagues’ (1438/1610, 90%), ‘learning as much as I can about COVID-19’ (1355/1619, 84%), ‘just accepting the inherent risk’ (1214/1605, 76%), and ‘trying not to think too much about the risks’ (1139/1610, 71%). Of note, 28% of respondents agreed that they used alcohol, marijuana, or other recreational drugs to cope with the stresses of the pandemic.

When asked about social media as a means to cope with the pandemic, more than half of respondents (852/1575, 54%) reported purposely avoiding social media, while 33% (314/962 SWO respondents) reported reading posts about COVID-19 on social media, and 17% (156/935 SWO respondents) reported posting about COVID-19 on social media.

### Supportive strategies

**Fig 5:**
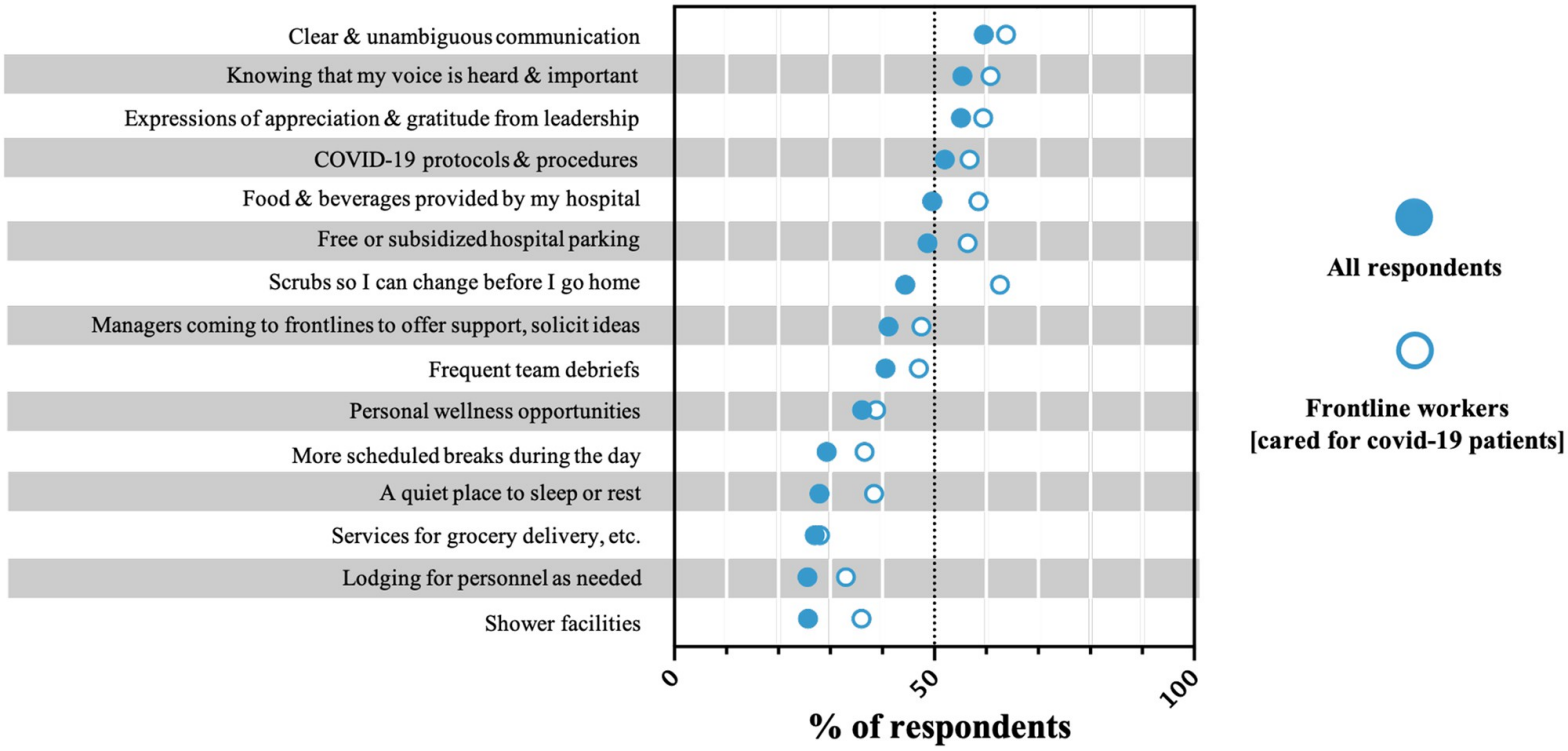
Preferred workplace strategies to help staff cope with the stresses of the COVID-19 pandemic. Data is presented separately for Frontline workers (unfilled circles) and All respondents (filled circles), which is inclusive of frontline and non-frontline workers to inform strategic implementation of supportive strategies either broadly (for all hospital workers) or targeted towards frontline workers only.

When asked to select strategies that would help them cope with the COVID-19 pandemic, the most frequently endorsed strategies included: clear and unambiguous communication from their hospital (59%), knowing that their voice is heard and important (55%), expressions of appreciation from hospital leadership (55%), having COVID-19 protocols and procedures (52%), and food and beverages provided by the hospital (50%). Fig 5 presents the proportion of respondents that selected each supportive strategy among all participants and among frontline workers only (those who reported caring for patients with suspected or confirmed COVID-19).

### Open-ended responses

Thematic content analysis of the open-ended questions identified 3 themes regarding the personal impact of the pandemic: (1) personal coping and wellness; (2) impact on family life; and (3) relationship with the community. The professional impact of the pandemic generated 5 themes related to: (1) changes in the work environment and activities; (2) concerns about patient care and wellbeing; (3) relationship with colleagues; (4) relationship with hospital leadership; and (5) PPE [S4 Table].

## Discussion

In this cross-sectional survey of hospital workers at 4 teaching and 8 non-teaching hospitals, we found that the COVID-19 pandemic has had important personal impact (e.g., fears and anxieties about exposure, falling ill, exposing others, stigmatization), professional impact (e.g., increased workload, workplace stress, expanded responsibilities), and psychological sequelae. One-quarter of respondents reported at least mild psychological distress on the K10 and nearly half had at least some symptoms of post-traumatic stress on the IES-R; 38% had scores that raise concern for at least one psychological diagnosis.

This study adds important information to the current paucity of data on the personal and professional impact of the COVID-19 pandemic on hospital workers. Overall, our findings are similar to those found in the aftermath of the SARS pandemic [2]. We found that a hospital staff continued their work in the pandemic setting despite believing that their chances of survival from COVID-19 were poor and that their chances of dying from COVID-19 within the next year are higher than that associated with a traffic collision or cancer. More than half of respondents reported stigmatization because of their work at a hospital, and over one-third had avoided telling people about the nature of their job. These rates are similar to a Singapore-based survey during the SARS pandemic, in which 49% HCWs reported that people had avoided them and 31% perceived that people had avoided their family members [2]. The most frequently reported personal coping strategies involved supportive relationships (i.e., talking to friends, family, and colleagues), informational (i.e., learning as much as I can about COVID-19), or attitudinal (i.e., accepting the inherent risk). Nevertheless, many respondents reported reluctance in telling their families about their exposure risk, which could adversely impact their coping. Of note, one-quarter of respondents endorsed use of ‘alcohol, marijuana, or other recreational drugs’ to cope with the stress of the pandemic and 44% contemplated leaving their job. These findings highlight the urgent need to identify HCWs at high risk of adverse outcomes, and to provide supportive strategies and ensure access to psychological counselling during and after the pandemic.

Similar to previous studies, nursing professional had more psychological distress than physicians [2, 7, 8]. The increased burden of psychological distress relative to physicians was also seen in other non-physician hospital workers (Fig 3; S3 Table), highlighting the need to ensure that support strategies implemented at the regional and hospital levels should be inclusive of nursing professionals and other health disciplines professionals. Other personal and professional characteristics have emerged as independent predictors of higher psychological symptoms during the COVID-19 pandemic: younger age [9, 11], female gender [7, 9, 10, 29], being a frontline worker [7, 12], and working in a community hospital [7, 9, 10, 12]. In this study, younger age and female gender were associated with higher workplace stress, psychological distress, as well as depression and anxiety symptoms, and female gender was also associated with higher post-traumatic stress symptoms. We also identified other factors associated with high workplace stress and psychological symptoms: having a high-risk health condition, personally knowing someone who contracted COVID-19, and feeling increased personal risk due to PPE shortage or inadequate PPE training. The latter finding highlights the importance of PPE supply and training in HCWs personal safety and perception of risk, and potentially mitigating the adverse effects of the pandemic.

Emerging evidence have shown that job insecurity and employee burnout during the COVID-19 pandemic influence customer orientation and workplace motivation, with broad implications for human resource management in sectors outside of healthcare [30]. Although job insecurity has not been a concern for healthcare workers as it has been for other workers during the COVID-19 pandemic, HCW burnout is certainly likely to influence workplace motivation and therefore patient care in the healthcare setting. Similar to non-healthcare settings, various organizational changes may mitigate the adverse effects of the COVID-19 pandemic on HCWs and the patient care they provide. In the present study involving employees in the healthcare sector, we identified several opportunities to create a supportive work environment that protects the wellbeing of hospital staff and fosters improved relationships in the workplace. The most frequently favoured supportive strategies were cost-free and primarily associated with the development of a more supportive culture by hospital leadership. These included clear and unambiguous communication from the hospital, HCWs knowing that their voice is heard and important, expressions of appreciation and gratitude from hospital leadership, and having COVID-19 protocols and procedures in place. These findings were also supported by the open-text responses, in which respondents expressed a great need to feel heard, understood, and appreciated by hospital management. Future studies should evaluate the impact of various supportive strategies in mitigating the adverse impact of pandemics on hospital workers.

This study has several limitations. We distributed the survey at a single timepoint and findings may not reflect evolving perspectives of workers as the pandemic persisted. It is not possible to determine an accurate response rate given that the survey was distributed through hospital-wide emails to all staff. More than 80% of respondents were female; while this is consistent with the demographics of the professions most highly represented in this survey (i.e., nursing professionals), the overall findings may best reflect the perspectives of women. Finally, hospital-wide distribution of the survey and inclusion of hospital staff with a broad range of professional characteristics disallows any conclusions about specific subgroups of hospital workers. This study has several strengths. First, we adapted a survey instrument that was used to evaluate the impact of the SARS pandemic on healthcare workers [5, 8], and administered two validated instruments evaluating psychological distress and symptoms of post-traumatic stress. We included a large sample of respondents representing a broad range of professions and practice settings (teaching and non-teaching) to ensure that the findings are generalizable and to inform supportive strategies applicable to a broad range of professions.

## Conclusion

In this cross-sectional survey of staff at 12 teaching and non-teaching hospitals, we found that the first wave of the COVID-19 pandemic had important personal, professional, and psychological effects on hospital workers. We identified several low-cost opportunities for healthcare systems and hospitals to support and address the needs of hospital workers during pandemics, including clear and unambiguous communication with staff, recognizing that the voice of HCWs is important, expressions of gratitude and appreciation by hospital leadership, and having COVID-19 protocols and procedures in place.

## Supporting information

S1 Appendix Checklist for Reporting Results of Internet E-Surveys (CHERRIES). (PDF)

S2 Appendix Survey instruments. (PDF)

S3 Appendix Media releases and email invitations to the survey. (PDF)

S1 Table Psychological instrument scoring. (PDF)

S2 Table Description of predictor variables associated with psychological burden. (PDF)

S3 Table Psychological distress, depression, anxiety, and post-traumatic stress symptoms among hospital workers during the COVID-19 pandemic by professional role. (PDF)

S4 Table Thematic consent analysis of open-ended responses. (PDF)

S1 Data (XLS)
