# Supplementary material for: Personal, professional, and psychological impact of the COVID-19 pandemic on hospital workers: A cross-sectional survey
Source: PLoS One. 2022 Feb 15;17(2):e0263438. doi: 10.1371/journal.pone.0263438 (PMC8846533; doi:10.1371/journal.pone.0263438)
Supplement: S1 Appendix — (PDF) [file pone.0263438.s001.pdf]

Appendix 1: Checklist for Reporting Results of Internet E-Surveys (CHERRIES)

Checklist Item	***Explanation***	***Description***	***Page Number***
Describe survey design	Describe target population, sample frame. Is the sample a convenience sample? (In "open" surveys this is most likely.)	Staff in 4 teaching and 8 non-teaching hospitals in Ontario, Canada	Abstract, 2<br>Methods, 5
IRB approval	Mention whether the study has been approved by an IRB.	The study was approved by the Sinai Health (20-0089-E) and Western University (#115850) Research Ethics Boards prior to survey distribution.	Methods, 5
Informed consent	Describe the informed consent process. Where were the participants told the length of time of the survey, which data were stored and where and for how long, who the investigator was, and the purpose of the study?	Consent was implied by completion of the survey.<br><br>Yes, participants were told the survey would take approximately 20 minutes to complete, who the investigator was and the purpose of the study.	Methods, 5<br><br>Appendix 1
Data protection	If any personal information was collected or stored, describe what mechanisms were used to protect unauthorized access.	Surveys were conducted using one of two secure, web-based platforms: NoviSurvey for Toronto sites and REDCap for Southwest Ontario sites.	Methods, 6
Development and testing	State how the survey was developed, including whether the usability and technical functionality of the electronic questionnaire had been tested before fielding the questionnaire.	We adapted a survey previously used during the SARS pandemic. Using formal survey development methodology, we iteratively refined the existing instrument and engaged representatives from stakeholder groups (nurses, physicians including a psychiatrist, allied professionals, and researchers with	Methods, 5

		pandemic expertise) for pre-testing to ensure that questions addressed concerns specific to hospital staff, and were likely to yield information pertinent to the study objective.	
Open survey versus closed survey	An “open survey” is a survey open for each visitor of a site, while a closed survey is only open to a sample which the investigator knows (password-protected survey).	Open survey	Methods, 6
Contact mode	Indicate whether or not the initial contact with the potential participants was made on the Internet. (Investigators may also send out questionnaires by mail and allow for Web-based data entry.)	Internet was the first point of contact with potential participants. Some participants were emailed a link to the survey while others could obtain the link from a COVID-19 research page on Toronto websites.	Methods, 6
Advertising the survey	How/where was the survey announced or advertised? Some examples are offline media (newspapers), or online (mailing lists – If yes, which ones?) or banner ads (Where were these banner ads posted and what did they look like?). It is important to know the wording of the announcement as it will heavily influence who chooses to participate.	Participants were invited via email by local hospital leadership to complete the survey, with 2 to 3 reminders over several weeks. In addition, the survey invitation and link were posted on the hospital COVID-19 research page for Toronto sites.	Methods, 6<br><br>Appendix 2

	Ideally the survey announcement should be published as an appendix.		
Web/E-mail	State the type of e-survey (eg, one posted on a Web site, or one sent out through e-mail). If it is an e-mail survey, were the responses entered manually into a database, or was there an automatic method for capturing responses?	The e-survey was posted on websites (NoviSurvey and REDCap).<br>Participants could access the e-survey via a link were posted on the hospital COVID-19 research page for Toronto sites or through email (sent a survey link by local hospital leadership).	Methods, 6
Context	Describe the Web site (for mailing list/newsgroup) in which the survey was posted. What is the Web site about, who is visiting it, what are visitors normally looking for? Discuss to what degree the content of the Web site could pre-select the sample or influence the results. For example, a survey about vaccination on a anti-immunization Web site will have different results from a Web survey conducted on a government Web site	The survey link was posted on a hospital based COVID-19 research page for Toronto centres. The website contained information about ongoing research with a focus on COVID-19 geared towards hospital staff.	Methods, 6
Mandatory/voluntary	Was it a mandatory survey to be filled in by every visitor who wanted to enter	Voluntary survey	Methods, 6

	the Web site, or was it a voluntary survey?		
Incentives	Were any incentives offered (eg, monetary, prizes, or non-monetary incentives such as an offer to provide the survey results)?	No.	N/A
Time/Date	In what timeframe were the data collected?	Data was collected over several weeks in July and September 2020.	Methods, 6
Randomization of items or questionnaires	To prevent biases items can be randomized or alternated.	Questions were not randomized as they were categorized and followed a logical order.	N/A
Adaptive questioning	Use adaptive questioning (certain items, or only conditionally displayed based on responses to other items) to reduce number and complexity of the questions.	Several items used adaptive questioning to reduce the number and frequency and complexity of the questions, based on demographic factors (i.e., some questions were specific to female respondents, some specific to those who indicated they had children, some specific to trainees, etc).	Not reported.
Number of Items	What was the number of questionnaire items per page? The number of items is an important factor for the completion rate.	The number of questions per page varied depending on the page and the responses to the adaptive questions.<br><br>Each page of the surveys contained approximately 10-25 individual items.	Not reported.
Number of screens (pages)	Over how many pages was the questionnaire distributed? The number of items is an important factor for the completion rate.	SWO survey: 19 pages (including Letter of information and consent)	Not reported.
Completeness check	It is technically possible to do consistency or completeness checks before	Participants were allowed to skip any question(s) and continue with the survey. As	Not reported.

	the questionnaire is submitted. Was this done, and if "yes", how (usually JAVAScript)? An alternative is to check for completeness after the questionnaire has been submitted (and highlight mandatory items). If this has been done, it should be reported. All items should provide a non-response option such as "not applicable" or "rather not say", and selection of one response option should be enforced.	such, a completeness check was not done before the questionnaire was submitted.	
Review step	State whether respondents were able to review and change their answers (eg, through a Back button or a Review step which displays a summary of the responses and asks the respondents if they are correct).	Yes.	Not reported.
Unique site visitor	If you provide view rates or participation rates, you need to define how you determined a unique visitor. There are different techniques available, based on IP addresses or cookies or both.	View rates not provided.	Not reported.

View rate (Ratio of unique survey visitors/unique site visitors)	Requires counting unique visitors to the first page of the survey, divided by the number of unique site visitors (not page views!). It is not unusual to have view rates of less than 0.1 % if the survey is voluntary.	We were unable to track the number of hospital website visitors as these data are not available to the researchers.	Not reported.
Participation rate (Ratio of unique visitors who agreed to participate/unique first survey page visitors)	Count the unique number of people who filled in the first survey page (or agreed to participate, for example by checking a checkbox), divided by visitors who visit the first page of the survey (or the informed consents page, if present). This can also be called “recruitment” rate.	We were unable to track the number of unique first page visitors, however, the number of visitors who proceeded beyond the consent page was 2097, of which 1875 completed items beyond the demographic questions and were included in the final analysis.	Not reported.
Completion rate (Ratio of users who finished the survey/users who agreed to participate)	The number of people submitting the last questionnaire page, divided by the number of people who agreed to participate (or submitted the first survey page). This is only relevant if there is a separate “informed consent” page or if the survey goes over several pages. This is a measure for attrition. Note that “completion” can involve	Participation rate was 89.4%. 2097 respondents accessed the survey, among which 1875 completed at least beyond the demographic questions and were included in the final analysis.	Not reported.

	leaving questionnaire items blank. This is not a measure for how completely questionnaires were filled in. (If you need a measure for this, use the word "completeness rate".)		
Cookies used	Indicate whether cookies were used to assign a unique user identifier to each client computer. If so, mention the page on which the cookie was set and read, and how long the cookie was valid. Were duplicate entries avoided by preventing users access to the survey twice; or were duplicate database entries having the same user ID eliminated before analysis? In the latter case, which entries were kept for analysis (eg, the first entry or the most recent)?	Cookies were not used to assign a unique user identifier.<br>REDCap, used for the SWO survey, uses a cookie for survey respondents taking surveys to store temporary data during the survey.	N/A
IP check	Indicate whether the IP address of the client computer was used to identify potential duplicate entries from the same user. If so, mention the period of time for which no two entries from the same IP	The IP address of the client computer was not used to identify potential duplicate entries from the same user. REDCap, used for the SWO survey, does employs "rate limiting" on its web pages, in which there is a set maximum number of web requests per minute that are allowed from a single IP address, and after that maximum is hit, the IP	N/A

	address were allowed (eg, 24 hours). Were duplicate entries avoided by preventing users with the same IP address access to the survey twice; or were duplicate database entries having the same IP address within a given period of time eliminated before analysis? If the latter, which entries were kept for analysis (eg, the first entry or the most recent)?	address of that user is permanently banned from REDCap. Rate limiting prevents denial of service attacks by bots as well as preventing other types of hacker attacks that require making many requests to the server in a short amount of time, such as with a BREACH attack.	
Log file analysis	Indicate whether other techniques to analyze the log file for identification of multiple entries were used. If so, please describe.	No other techniques were used to analyze the log file for identification of multiple entries.	N/A
Registration	In “closed” (non-open) surveys, users need to login first and it is easier to prevent duplicate entries from the same user. Describe how this was done. For example, was the survey never displayed a second time once the user had filled it in, or was the username stored together with the survey results and later eliminated? If the latter, which entries were	This was an “open” survey.	N/A

	kept for analysis (eg, the first entry or the most recent)?		
Handling of incomplete questionnaires	Were only completed questionnaires analyzed? Were questionnaires which terminated early (where, for example, users did not go through all questionnaire pages) also analyzed?	Questionnaires that were terminated early were analyzed as long as the participant completed items beyond the demographic questions (i.e., responded to questions about COVID-19 exposure and beyond).	Results, 7
Questionnaires submitted with an atypical timestamp	Some investigators may measure the time people needed to fill in a questionnaire and exclude questionnaires that were submitted too soon. Specify the timeframe that was used as a cut-off point, and describe how this point was determined.	We did not exclude questionnaires based on any criteria related to timestamp.	N/A
Statistical correction	Indicate whether any methods such as weighting of items or propensity scores have been used to adjust for the non-representative sample; if so, please describe the methods.	No statistical correction for non-representative sample as we consider the sample to be representative of the hospital workers in the regions surveyed.	N/A

This checklist has been modified from Eysenbach G. Improving the quality of Web surveys: the Checklist for Reporting Results of Internet E-Surveys (CHERRIES). J Med Internet Res. 2004 Sep 29;6(3):e34 [erratum in J Med Internet Res. 2012; 14(1): e8.]. Article available at

https://www.jmir.org/2004/3/e34/; erratum available https://www.jmir.org/2012/1/e8/. Copyright ©Gunther Eysenbach. Originally published in the Journal of Medical Internet Research, 29.9.2004 and 04.01.2012.

This is an open-access article distributed under the terms of the Creative Commons Attribution License (https://creativecommons.org/licenses/by/2.0/), which permits unrestricted use, distribution, and reproduction in any medium, provided the original work, first published in the Journal of Medical Internet Research, is properly cited.
