# Supplementary material for: Personal, professional, and psychological impact of the COVID-19 pandemic on hospital workers: A cross-sectional survey
Source: PLoS One. 2022 Feb 15;17(2):e0263438. doi: 10.1371/journal.pone.0263438 (PMC8846533; doi:10.1371/journal.pone.0263438)
Supplement: S2 Appendix — (PDF) [file pone.0263438.s002.pdf]

Appendix 2: Survey Instruments

A survey of the personal and professional impact of COVID-19 on health care practitioners and support staff at Sinai Health System

We invite you to participate in a questionnaire research study of the effects of the COVID-19 pandemic on health care practitioners (HCPs) and essential support staff at Sinai Health.

Hospitals across Canada are currently providing care for COVID-19 patients, or are involved in pandemic preparedness. We are faced with a rapidly changing situation and the barrage of news and social media can be overwhelming and confusing. We are all experiencing a range of emotions.

The purpose of this survey is to evaluate the work-related and non-work related concerns regarding the COVID-19 outbreak of those who work at Mount Sinai Hospital and Bridgepoint. We seek input from nurses, physicians, respiratory therapists, pharmacists, clerical staff, allied health, environmental services, and any other hospital personnel.

The aims of this study are to describe the effects of the COVID-19 pandemic (and preparations for it) on:

o Your work in the hospital

o Concerns about yourself and your family members

o Anxiety, stress, and your mental health

o Any strategies that have helped you cope

The survey will take approximately 20 minutes to complete. If you agree to participate, please click on the link below.

If you prefer to complete the survey on paper, please collect a paper copy of the survey from an envelope in your department and drop the completed survey back at the same place in an envelope with a completed survey sign.

Please complete the survey once only, either on paper or online but not both.

Participation is voluntary and submitting data implies your consent to participate. We encourage you to respond to all questions, but you can skip any questions and still continue with the survey. The information you provide will be kept anonymous and confidential.

Questions? Please email Geeta.mehta@sinaihealth.ca

If you have any questions about your rights as a research participant or have concerns about this study, call Ronald Heslegrave, PhD, Chair of the Mount Sinai Hospital Research Ethics Board (REB) or the Research Ethics office number at 416-586-4875, during regular business hours (9:00 am to 5:00 pm). The REB is a group of people who oversee the ethical conduct of research studies. These people are not part of the study team. Everything that you discuss will be kept confidential.

Thank you.

Geeta Mehta MD, Christopher Yarnell MD, Jennie Johnstone MD PhD, Chaim Bell MD, Lianne Jeffs RN PhD FAAN, Alex Kiss PhD, and Robert Maunder MD

The Impact of COVID-19 on Sinai Health System

A. Personal Details

1. I work at (select one or both) ☐ Mount Sinai Hospital ☐ Bridgepoint

2. My job type is (select one response only)

☐ MD - Staff Physician ☐ MD - Fellow ☐ MD - Resident ☐ MD - Student
☐ Nurse ☐ Nurse Aide ☐ Respiratory Therapy ☐ Pharmacy
☐ Dietetics ☐ Physiotherapy ☐ Occupational Therapy ☐ Speech Language Pathology
☐ Social Work ☐ Spiritual Care ☐ Research ☐ Manager
☐ Engineering ☐ Housekeeping ☐ Food Services ☐ Clerical
☐ Laboratory Services ☐ Security ☐ Porter ☐ Volunteer
☐ Radiology technologist ☐ Echo technologist ☐ IT/ Information technology
☐ Student (e.g. Nursing, Respiratory Therapy, Pharmacy, Physiotherapy) ☐ Other ___________________

3. What is your clinical area?

☐ Cardiology ☐ Emergency Department ☐ ENT Surgery
☐ General Internal Medicine ☐ General Surgery ☐ Intensive care unit
☐ Neonatal ICU ☐ Obstetrics / Gynecology ☐ Orthopedic Surgery
☐ Psychiatry ☐ Radiology ☐ Other ___________________________
☐ N/A – I don't work in a clinical area

4. My age is ☐ < 30 yrs ☐ 31-40 ☐ 41-50 ☐ 51-60 ☐ 61-70 ☐ > 70 yrs

5. The length of my experience as a hospital/health care worker is:

☐ ≤ 5 years ☐ 6-10 years ☐ 11-20 years ☐ > 20 years

6. My gender is ☐ Male ☐ Female ☐ Non-binary ☐ Prefer not to answer

7. (if yes to 'Female') Are you pregnant or do you think you might be pregnant? ☐ Yes ☐ No

8. Do you take medication or have a medical condition that places you at higher risk of poor outcomes if you were infected with COVID-19? ☐ Yes ☐ No

9. My ethnic group is

☐ Indigenous (First Nations, Inuit, Metis, person from any country)
☐ Black
☐ East Asian (Chinese, Japanese, Korean etc.)
☐ South Asian (Indian, Pakistani, Sri Lankan, East Indian from Guyana, etc.)
☐ Southeast Asian (Filipino, Cambodian, Indonesian, Laotian, etc.)
☐ West Asian (Iranian, Iraqi, Persian, etc)
☐ Central Asian (Kazakh, Afghan, Tajik, etc.)
☐ Middle Eastern
☐ White
☐ Other (specify): ______________
☐ I prefer not to answer

10. My marital status is ☐ Single ☐ Married/common-law ☐ Divorced/Separated ☐ Widowed ☐ I prefer not to answer

11. Do you have children? ☐ Yes ☐ No

(if 'yes') The number of children I have is ________. What are their ages? #1______#2______#3_______#4______#5________

The number of children living with me is _________. What are their ages? #1______#2______#3______#4______#5_____

I am a single parent ☐ Yes ☐ No

12. I am presently living with (check all that apply)
☐ my partner
☐ my child/children
☐ extended family (e.g., parents, siblings, grandchildren)
☐ someone > 65 years
☐ someone who is immunocompromised
☐ roommate(s)
☐ caregiver / nanny
☐ I live alone☐ Other (please specify)_________________________

13. The nature of my work since the COVID-19 outbreak has involved direct daily contact with (check all that apply)
☐ Suspected or confirmed COVID-19 patients
☐ Non-COVID-19 patients
☐ Members of the public (e.g., hospital visitors)
☐ None of the above

14. I think I have been exposed to COVID-19 in the hospital
☐ Daily ☐ Few times per week ☐ Rarely ☐ Not at all ☐ Don't know

15. I personally know people who are COVID-19 positive who are my (check all that apply)
☐ Immediate family members ☐ Close friends ☐ Community members
☐ Colleagues ☐ Acquaintances ☐ I personally don't know any COVID-19 patients

16. Check ALL of the following that apply to you.
☐ COVID-19 patients have been treated in my clinical area
☐ I have personally attended to patients in isolation for confirmed COVID-19
☐ I have personally attended to patients in isolation for suspected COVID-19
☐ I have been in quarantine because of possible exposure to COVID-19
☐ I have been in quarantine because of a travel history
☐ I have personally had close contact with COVID-19 patients outside of my work
☐ Someone living in my home has confirmed COVID-19
☐ I have been asked to stay at home due to "non-essential" status
☐ I have been separated from my family because of COVID-19
☐ I have worked in the screening process for staff, patients or visitors
☐ I have participated in the planning and preparations for COVID-19

SD=strongly disagree, D=disagree, PD=not sure but probably disagree, PA=not sure but probably agree, A=agree, SA=strongly agree, NA=not applicable

B. Exposure to COVID-19, Perception of Risk and Coping

	SD	D	PD	PA	A	SA	NA
**With regard to the risk of contracting COVID-19 myself**							
17. At my place of work, I believe that I have been at high risk of getting COVID-19 from:							
a. Close contact with COVID-19 patients	☐	☐	☐	☐	☐	☐	☐
b. The air that I breathe	☐	☐	☐	☐	☐	☐	☐
c. Close contact with colleagues exposed to COVID-19 patients	☐	☐	☐	☐	☐	☐	☐
d. Objects and surfaces	☐	☐	☐	☐	☐	☐	☐
e. Shortage of personal protective equipment (PPE)	☐	☐	☐	☐	☐	☐	☐
f. Inadequate training to use PPE	☐	☐	☐	☐	☐	☐	☐
18. I feel that my job has put me at great risk of exposure to COVID-19	☐	☐	☐	☐	☐	☐	☐
19. I have been afraid of falling ill with COVID-19	☐	☐	☐	☐	☐	☐	☐
20. I feel that I have had little control over whether I get infected or not	☐	☐	☐	☐	☐	☐	☐
21. I feel that I shouldn't have been looking after COVID-19 patients	☐	☐	☐	☐	☐	☐	☐
22. I have accepted the risk of getting COVID-19 as part of my job	☐	☐	☐	☐	☐	☐	☐
23. I am confident my employer would look after my medical needs if I were to fall ill with COVID-19	☐	☐	☐	☐	☐	☐	☐
24. My family believes that I have a high risk of getting COVID-19	☐	☐	☐	☐	☐	☐	☐
25. If I were to get COVID-19 I believe that my chances of survival are poor	☐	☐	☐	☐	☐	☐	☐
26. I believe that my chance of dying from COVID-19 over the next year is higher than dying from a. a traffic accident	☐	☐	☐	☐	☐	☐	☐
b. cancer	☐	☐	☐	☐	☐	☐	☐
27. I feel that people close to me outside of my work have been at high risk of getting COVID-19 because of my job	☐	☐	☐	☐	☐	☐	☐
28. I have been concerned about passing COVID-19 to							
a. My family members	☐	☐	☐	☐	☐	☐	☐
b. Close friends	☐	☐	☐	☐	☐	☐	☐
c. Work colleagues	☐	☐	☐	☐	☐	☐	☐
d. My patients	☐	☐	☐	☐	☐	☐	☐
e. Others	☐	☐	☐	☐	☐	☐	☐
29. People close to me have been worried about my health	☐	☐	☐	☐	☐	☐	☐
30. People close to me have been worried that they might get infected through me	☐	☐	☐	☐	☐	☐	☐
31. I have been preoccupied with my own symptoms and sensations, such as feeling hot or coughing	☐	☐	☐	☐	☐	☐	☐
32. It has been hard to feel reassured that I am healthy	☐	☐	☐	☐	☐	☐	☐
**I have personally coped with the COVID-19 situation by:**							
33. Learning as much as I can about COVID-19	☐	☐	☐	☐	☐	☐	☐
34. Avoiding reading too much about COVID-19	☐	☐	☐	☐	☐	☐	☐
35. Avoiding social media	☐	☐	☐	☐	☐	☐	☐
36. Taking nutritional supplements, vitamins and tonics	☐	☐	☐	☐	☐	☐	☐
37. Adhering to protocols and recommended measures	☐	☐	☐	☐	☐	☐	☐
38. Just accepting the inherent risk	☐	☐	☐	☐	☐	☐	☐
39. Trying not to think too much about the risks	☐	☐	☐	☐	☐	☐	☐
40. Keeping my mind positive and convincing myself that I won't develop COVID-19	☐	☐	☐	☐	☐	☐	☐
41. Avoiding crowded places	☐	☐	☐	☐	☐	☐	☐
42. Avoiding colleagues who may be exposed	☐	☐	☐	☐	☐	☐	☐
43. Exercising regularly	☐	☐	☐	☐	☐	☐	☐
44. Talking to friends, family and colleagues	☐	☐	☐	☐	☐	☐	☐
45. Using alcohol, marijuana or other recreational drugs	☐	☐	☐	☐	☐	☐	☐
46. Cleaning my environment (car, home, etc)	☐	☐	☐	☐	☐	☐	☐

SD=strongly disagree, D=disagree, PD=not sure but probably disagree, PA=not sure but **probably agree, A=agree,** SA=strongly agree, NA=not applicable

**I believe that the following measures are useful in protecting me from contracting COVID-19**	SD	D	PD	PA	A	SA	NA
47. Availability of occupational health service	☐	☐	☐	☐	☐	☐	☐
48. Screening of patients and hospital visitors for symptoms	☐	☐	☐	☐	☐	☐	☐
49. Limiting number of visitors to hospital	☐	☐	☐	☐	☐	☐	☐
50. Enforcing working from home for non-essential staff	☐	☐	☐	☐	☐	☐	☐
51. Information provided by my hospital	☐	☐	☐	☐	☐	☐	☐
52. The personalized mask-fit testing at my hospital	☐	☐	☐	☐	☐	☐	☐
53. Use of face protection (surgical masks, N95 masks, face shields)	☐	☐	☐	☐	☐	☐	☐
54. Gowns	☐	☐	☐	☐	☐	☐	☐
55. Hair cover	☐	☐	☐	☐	☐	☐	☐
56. Gloves	☐	☐	☐	☐	☐	☐	☐
57. Alcohol hand rub	☐	☐	☐	☐	☐	☐	☐
58. Regular hand washing	☐	☐	☐	☐	☐	☐	☐
59. Isolation of COVID-19 patients	☐	☐	☐	☐	☐	☐	☐

**With regard to the implementation of protective measures at work**							
60. I feel that they are generally effective	☐	☐	☐	☐	☐	☐	☐
61. I am satisfied with the explanation of their necessity and importance	☐	☐	☐	☐	☐	☐	☐
62. I feel that I received adequate training in which PPE (mask, faceshield, gloves, gown) to use	☐	☐	☐	☐	☐	☐	☐
63. I feel that I received adequate training on the safe removal of PPE	☐	☐	☐	☐	☐	☐	☐
64. I have someone to turn to when I have a problem using equipment	☐	☐	☐	☐	☐	☐	☐
65. Emotional support (e.g., counselling) is available to those who need help	☐	☐	☐	☐	☐	☐	☐
66. I feel that the policies and protocols instituted for us to follow are clear	☐	☐	☐	☐	☐	☐	☐
67. The information and instructions provided to us are changing too rapidly to keep up	☐	☐	☐	☐	☐	☐	☐
68. I feel that the policies and protocols were implemented quickly enough	☐	☐	☐	☐	☐	☐	☐
69. I think that most staff adhere to recommended measures consistently	☐	☐	☐	☐	☐	☐	☐
70. I have little difficulty in adhering to recommended measures	☐	☐	☐	☐	☐	☐	☐
71. I feel that healthcare workers treating COVID-19 patients deserve a higher level of personal protection than currently provided	☐	☐	☐	☐	☐	☐	☐

**<u>C. Impact of the COVID-19 epidemic on personal life and work</u>**							
72. I have someone to talk to and confide in about my concerns	☐	☐	☐	☐	☐	☐	☐
73. I have been afraid to tell my family about the risk I am exposed to	☐	☐	☐	☐	☐	☐	☐
74. I think people have avoided me because of my job	☐	☐	☐	☐	☐	☐	☐
75. I think people have avoided my family members because of my job	☐	☐	☐	☐	☐	☐	☐
76. I have avoided telling other people about the nature of my job	☐	☐	☐	☐	☐	☐	☐
77. I feel appreciated by the hospital/my employer	☐	☐	☐	☐	☐	☐	☐
78. I feel appreciated by society	☐	☐	☐	☐	☐	☐	☐
79. I think that the morale at work has been good	☐	☐	☐	☐	☐	☐	☐
80. I feel that there is adequate staff at my workplace	☐	☐	☐	☐	☐	☐	☐
81. I feel that there is more tension and conflict amongst colleagues at work	☐	☐	☐	☐	☐	☐	☐
82. I have felt more stressed at work	☐	☐	☐	☐	☐	☐	☐
83. I think that I have had an increase in workload	☐	☐	☐	☐	☐	☐	☐
84. I have had to work overtime	☐	☐	☐	☐	☐	☐	☐
85. I have had to do work that I normally don't do	☐	☐	☐	☐	☐	☐	☐
86. I have thought about leaving my job	☐	☐	☐	☐	☐	☐	☐
87. School closures have meant that I have to stay home to provide childcare	☐	☐	☐	☐	☐	☐	☐

D. Which of the following strategies would help you cope or are helping you cope at this time? (select all that apply)

- ☐ Opportunities to learn/practice personal wellness (e.g. mindfulness, meditation)
- ☐ Mindfulness Apps like “Headspace”
- ☐ Prayer
- ☐ Managers coming to the “front lines” to offer support and solicit ideas
- ☐ Expressions of appreciation and gratitude from my hospital leadership
- ☐ Knowing my voice is heard and is important
- ☐ Being part of COVID-19 planning at my hospital
- ☐ Clear and unambiguous communication about COVID-19 from my hospital
- ☐ Less communication about COVID-19 from my hospital
- ☐ A help phoneline for emotional support
- ☐ Having protocols and procedures about COVID-19
- ☐ Frequent team debriefs
- ☐ More frequent scheduled breaks throughout the day
- ☐ Breaks from caring for COVID-19 patients, for several days
- ☐ A quiet place to sleep or rest in the hospital
- ☐ Food and beverages provided by the hospital
- ☐ Scrubs provided by the hospital so I can change my clothes before I go home
- ☐ Shower facilities in the hospital so I can shower before I go home
- ☐ Lodging for personnel who live far away from the hospital, or for those who become infected
- ☐ Transportation to /from the hospital
- ☐ Free or subsidized hospital parking
- ☐ Childcare
- ☐ Eldercare
- ☐ Service for grocery delivery, etc
- ☐ Other (please write your suggestions)____

THANK YOU for completing this questionnaire!

Questionnaire- Impact of COVID-19 on Healthcare workers in South West Ontario

Professional Information

My primary hospital is... ______________________________

My primary job type is...
[please select one response]

◯ MD - Staff Physician
◯ MD - Fellow
◯ MD - Resident
◯ MD - Student
◯ Nurse
◯ Nurse Aide
◯ Respiratory Therapy
◯ Occupational Therapy
◯ Physiotherapy
◯ Pharmacy
◯ Dietetics
◯ Speech Language Pathology
◯ Social Work
◯ Spiritual Care
◯ Manager
◯ Clerical
◯ Research
◯ Porter
◯ Volunteer
◯ Environmental Assistant or Housekeeping
◯ Security
◯ Engineering
◯ Food Services
◯ Laboratory Services
◯ Radiology Technologist
◯ Echocardiography technologist
◯ Information Technology
◯ Student (please specify)
◯ Other (please specify)

Please specify what type of student
[e.g., Nursing, Respiratory Therapy, Pharmacy, Physiotherapy] ______________________________

Please specify your primary job type. ______________________________

Do you work in a clinical area of the hospital?

◯ Yes
◯ No

I work in a clinical area that cares for...<br>[please select all that apply]	☐ Adult patients<br>☐ Pediatric patients<br>☐ Neonatal patients
What is your usual clinical area(s)?	☐ Emergency Department<br>☐ General Internal Medicine<br>☐ Intensive care unit<br>☐ Cardiology<br>☐ Respirology<br>☐ Psychiatry<br>☐ Obstetrics/ Gynecology<br>☐ General Surgery<br>☐ Orthopedic Surgery<br>☐ ENT Surgery<br>☐ Radiology<br>☐ Other<br>☐ I don't work in a clinical area
Please specify your primary clinical area.	__________________________
The length of my experience as a hospital/ healthcare worker is...	◯ ≤ 5 years<br>◯ 6-10 years<br>◯ 11-20 years<br>◯ > 20 years
Were you deployed to another clinical area within the past 3 to 4 months [i.e., during the COVID-19 pandemic]?	☐ Yes<br>☐ No
What is your new clinical area(s)?	☐ Emergency Department<br>☐ General Internal Medicine<br>☐ Intensive care unit<br>☐ Cardiology<br>☐ Respirology<br>☐ Psychiatry<br>☐ Obstetrics/ Gynecology<br>☐ General Surgery<br>☐ Orthopedic Surgery<br>☐ ENT Surgery<br>☐ Radiology<br>☐ Other<br>☐ I don't work in a clinical area
Please specify the particular clinical area.	__________________________

10% Complete

Demographic Information

My age is...

◯ ≤ 30 ◯ 31-40 ◯ 41-50 ◯ 51-60 ◯ 61-70 ◯ >70

My gender is...

◯ Male ◯ Female ◯ Non-binary ◯ Prefer not to answer

Are you pregnant or do you think you might be pregnant?

◯ Yes ◯ No

My marital status is...

◯ Single ◯ Married/Common law ◯ Divorced/Separated ◯ Widowed ◯ I prefer not to answer

My ethnic group[s] is/ are...
[please select all that apply]

- ☐ Indigenous (First Nations, Inuit, Metis, person from any country)
- ☐ Black
- ☐ East Asian (Chinese, Japanese, Korean etc.)
- ☐ South Asian (Indian, Pakistani, Sri Lankan, East Indian from Guyana, etc.)
- ☐ Southeast Asian (Filipino, Cambodian, Indonesian, Laotian, etc.)
- ☐ West Asian (Iranian, Iraqi, Persian, etc)
- ☐ Central Asian (Kazakh, Afghan, Tajik, etc.)
- ☐ Middle Eastern
- ☐ White
- ☐ Other (specify): ______________
- ☐ I prefer not to answer

Please specify your ethnic group[s].

____

Do you take medication or have a medical condition that places you at higher risk of poor outcomes if you were infected with COVID-19?

◯ Yes ◯ No

Do you have children?

◯ Yes ◯ No

The number of children I have is...

____

What are their ages?
[please list the ages of your children in this box]

I am a single parent.

◯ Yes ◯ No

I am presently living with [please select all that apply]

☐ My partner/ spouse
☐ My child/ children
☐ Extended family (e.g., parents, siblings, grandchildren)
☐ Someone > 65 years
☐ Someone who is immunocompromised
☐ Roommate(s)
☐ Caregiver/Nanny
☐ I live alone
☐ Other

Please specify who you are presently living with.

____

25% Complete

COVID-19 Exposures

COVID has impacted my personal life because [please select all that apply]	☐ I have contracted COVID-19<br>☐ I have personally had close contact with COVID-19 patients outside of my work<br>☐ Someone living in my home has confirmed COVID-19<br>☐ I have been separated from my family because of COVID-19<br>☐ COVID-19 has not impacted my personal life
The nature of my work since the COVID-19 outbreak has involved direct contact with [please select all that apply]	☐ Suspected or confirmed COVID-19 patients<br>☐ Non-Covid-19 patients<br>☐ Members of the public (e.g., hospital visitors)<br>☐ None of the above
Please check all of the following that apply to you.	☐ COVID-19 patients have been treated in my clinical area<br>☐ I have personally attended to patients in isolation for confirmed COVID-19<br>☐ I have personally attended to patients in isolation for suspected COVID-19<br>☐ I have been in quarantine because of possible exposure to COVID-19<br>☐ I have been in quarantine because of a travel history<br>☐ I have personally had close contact with COVID-19 patients outside of my work<br>☐ Someone living in my home has confirmed COVID-19<br>☐ I have been asked to stay at home due to "non-essential" status<br>☐ I have been separated from my family because of COVID-19<br>☐ I have worked in the screening process for staff, patients or visitors<br>☐ I have participated in the planning and preparations for COVID-19
I think I have been exposed to COVID-19 in the hospital...	○ Daily<br>○ Few times per week<br>○ Rarely<br>○ Not at all<br>○ Don't know

35% Complete

Risk of Exposure to COVID-19

I believe that I have been at high risk of getting COVID-19 from...

	Strongly Disagree	Disagree	Not Sure But Probably Disagree	Not Sure But Probably Agree	Agree	Strongly Agree	Not Applicable
Close contact with COVID-19 patients	○	○	○	○	○	○	○
The air that I breathe	○	○	○	○	○	○	○
Close contact with colleagues exposed to COVID-19 patients	○	○	○	○	○	○	○
Objects and surfaces	○	○	○	○	○	○	○
Shortage of personal protective equipment (PPE)	○	○	○	○	○	○	○
Inadequate training to use PPE	○	○	○	○	○	○	○

Risk of Exposure to COVID-19

With regard to the risk of contracting COVID-19 myself...

	Strongly Disagree	Disagree	Not Sure But Probably Disagree	Not Sure But Probably Agree	Agree	Strongly Agree	Not Applicable
I feel that my job has put me at great risk of exposure to COVID-19	○	○	○	○	○	○	○
I have been afraid of falling ill with COVID-19	○	○	○	○	○	○	○
I feel that I have had little control over whether I get infected or not	○	○	○	○	○	○	○
I feel that I shouldn't have been looking after COVID-19 patients	○	○	○	○	○	○	○
I have accepted the risk of getting COVID-19 as part of my job	○	○	○	○	○	○	○
I am confident my employer would look after my medical needs if I were to fall ill with COVID-19	○	○	○	○	○	○	○
My family believes that I have a high risk of getting COVID-19	○	○	○	○	○	○	○
If I were to get COVID-19 I believe that my chances of survival are poor	○	○	○	○	○	○	○
People close to me have been worried about my health	○	○	○	○	○	○	○
People close to me have been worried that they might get infected through me	○	○	○	○	○	○	○
I have been preoccupied with my own symptoms and sensations, such as feeling hot or coughing	○	○	○	○	○	○	○
It has been hard to feel reassured that I am healthy	○	○	○	○	○	○	○
I feel that people close to me outside of my work have been at high risk of getting COVID-19 because of my job	○	○	○	○	○	○	○

I believe that my chance of dying from COVID-19 over the next year is higher than dying from...

	Strongly Disagree	Disagree	Not Sure But Probably Disagree	Not Sure But Probably Agree	Agree	Strongly Agree	Not Applicable
A traffic accident	○	○	○	○	○	○	○
Cancer	○	○	○	○	○	○	○

Please indicate your level of agreement with the following statements.

	Strongly Disagree	Disagree	Not Sure But Probably Disagree	Not Sure But Probably Agree	Agree	Strongly Agree	Not Applicable
I am concerned about the effect of the COVID-19 pandemic on patient care	○	○	○	○	○	○	○
I am concerned that restrictions on hospital visitors (i.e., families) will have a negative effect on my patients	○	○	○	○	○	○	○
I am concerned that I have not been adequately trained to manage the patients I have been asked to care for	○	○	○	○	○	○	○
I am concerned about passing COVID-19 to my family members	○	○	○	○	○	○	○
I am concerned about passing COVID-19 to my close friends	○	○	○	○	○	○	○
I am concerned about passing COVID-19 to my patients	○	○	○	○	○	○	○
I am concerned about passing COVID-19 to my work colleagues	○	○	○	○	○	○	○
I am concerned about passing COVID-19 to others	○	○	○	○	○	○	○

Please specify which other groups you are concerned of passing COVID-19 to. ______________________________

Impact of the Pandemic

Regarding the impact of the COVID-19 pandemic on your personal life & work, please indicate your agreement with the following statements.

	Strongly Disagree	Disagree	Not Sure But Probably Disagree	Not Sure But Probably Agree	Agree	Strongly Agree	Not Applicable
I have someone to talk to and confide in about my concerns	○	○	○	○	○	○	○
I have been afraid to tell my family about the risk I am exposed to	○	○	○	○	○	○	○
I think people have avoided me because of my job	○	○	○	○	○	○	○
I think people have avoided my family members because of my job	○	○	○	○	○	○	○
I have avoided telling other people about the nature of my job	○	○	○	○	○	○	○
I feel appreciated by the hospital/my employer	○	○	○	○	○	○	○
I feel appreciated by society	○	○	○	○	○	○	○
I think that the morale at work has been good	○	○	○	○	○	○	○
I feel that there is more tension and conflict amongst colleagues at work	○	○	○	○	○	○	○
I have felt more stressed at work	○	○	○	○	○	○	○
I think that I have had an increase in workload	○	○	○	○	○	○	○
I have had to work overtime	○	○	○	○	○	○	○
I have had to do work that I normally don't do	○	○	○	○	○	○	○
I have thought about leaving my job	○	○	○	○	○	○	○
School closures have meant that I have to stay home to provide childcare	○	○	○	○	○	○	○

Protective Measures

I believe that the following measures are useful in protecting me from contracting COVID-19.

	Strongly Disagree	Disagree	Not Sure But Probably Disagree	Not Sure But Probably Agree	Agree	Strongly Agree	Not Applicable
Availability of occupational health service	○	○	○	○	○	○	○
Screening of patients and hospital visitors for symptoms	○	○	○	○	○	○	○
Screening of hospital staff for symptoms	○	○	○	○	○	○	○
Limiting number of visitors to hospital	○	○	○	○	○	○	○
Enforcing working from home for non-essential staff	○	○	○	○	○	○	○
Information provided by my hospital	○	○	○	○	○	○	○
The personalized mask-fit testing at my hospital	○	○	○	○	○	○	○
Use of face protection [surgical masks, N95 masks, face shields]	○	○	○	○	○	○	○
Gowns	○	○	○	○	○	○	○
Hair cover	○	○	○	○	○	○	○
Gloves	○	○	○	○	○	○	○
Alcohol hand sanitizer	○	○	○	○	○	○	○
Regular hand washing	○	○	○	○	○	○	○
Isolation of COVID-19 patients	○	○	○	○	○	○	○

Protective Measures

With regards to the implementation of protective measures at work...

	Strongly Disagree	Disagree	Not Sure But Probably Disagree	Not Sure But Probably Agree	Agree	Strongly Agree	Not Applicable
I feel that PPEs are generally effective	○	○	○	○	○	○	○
I am satisfied with the explanation of the necessity and importance of PPE use	○	○	○	○	○	○	○
I feel that I received adequate training in which PPE [i.e., mask type, face shield, gloves, gown] to use	○	○	○	○	○	○	○
I feel that I received adequate training on the safe removal of PPE	○	○	○	○	○	○	○
I have someone to turn to when I have a problem using equipment	○	○	○	○	○	○	○
Emotional support [e.g. counselling] is available to those who need help	○	○	○	○	○	○	○
I feel that the policies and protocols instituted for us to follow are clear	○	○	○	○	○	○	○
The information and instructions provided to us are changing too rapidly to keep up	○	○	○	○	○	○	○
I feel that the policies and protocols were implemented quickly enough	○	○	○	○	○	○	○
I think that most staff adhere to recommended measures consistently	○	○	○	○	○	○	○
I have little difficulty in adhering to recommended measures	○	○	○	○	○	○	○
I feel that healthcare workers treating COVID-19 patients deserve a higher level of personal protection than currently provided	○	○	○	○	○	○	○

Coping Strategies

I have personally coped with the COVID-19 situation by...

	Strongly Disagree	Disagree	Not Sure But Probably Disagree	Not Sure But Probably Agree	Agree	Strongly Agree	Not Applicable
Learning as much as I can about COVID-19	○	○	○	○	○	○	○
Avoiding reading too much about COVID-19	○	○	○	○	○	○	○
Reading posts about COVID-19 on social media	○	○	○	○	○	○	○
Writing or sharing posts about COVID-19 on social media	○	○	○	○	○	○	○
Avoiding social media	○	○	○	○	○	○	○
Taking nutritional supplements, vitamins and tonics	○	○	○	○	○	○	○
Adhering to protocols and recommended measures	○	○	○	○	○	○	○
Just accepting the inherent risk	○	○	○	○	○	○	○
Trying not to think too much about the risks	○	○	○	○	○	○	○
Keeping my mind positive and convincing myself that I won't develop COVID-19	○	○	○	○	○	○	○
Avoiding crowded places	○	○	○	○	○	○	○
Avoiding colleagues who may be exposed	○	○	○	○	○	○	○
Exercising regularly	○	○	○	○	○	○	○
Talking to friends, family, and colleagues	○	○	○	○	○	○	○
Using alcohol, marijuana, or other recreational drugs	○	○	○	○	○	○	○
Cleaning my environment [e.g., car, home]	○	○	○	○	○	○	○
Using mindfulness Apps like 'Headspace'	○	○	○	○	○	○	○
Prayer	○	○	○	○	○	○	○

Which of the following strategies would help you cope or are helping you cope at this time? [please select all that apply]

☐ Opportunities to learn/practice personal wellness (e.g. mindfulness, meditation)
☐ Managers coming to the "front lines" to offer support and solicit ideas
☐ Expressions of appreciation and gratitude from my hospital leadership
☐ Knowing my voice is heard and is important
☐ Being part of COVID-19 pandemic planning at my hospital
☐ Clear and unambiguous communication about COVID-19 from my hospital
☐ Less communication about COVID-19 from my hospital
☐ A help phoneline for emotional support
☐ Having protocols and procedures about COVID-19
☐ Frequent team debriefs
☐ More frequent scheduled breaks throughout the day
☐ Breaks from caring for COVID-19 patients, for several days
☐ A quiet place to sleep or rest in the hospital
☐ Food and beverages provided by the hospital
☐ Scrubs provided by the hospital so I can change my clothes before I go home
☐ Shower facilities in the hospital so I can shower before I go home
☐ Lodging for personnel who live far away from the hospital, or for those who become infected
☐ Transportation to /from the hospital
☐ Free or subsidized hospital parking
☐ Childcare
☐ Eldercare
☐ Services such as grocery delivery, etc
☐ Other

Please write your suggestion[s].

____

55% Complete

Below is a list of difficulties people sometimes have after stressful life events. How much were you distressed or bothered by these difficulties during the COVID-19 pandemic?

	Not at all	A little bit	Moderately	Quite a bit	Extremely
Any reminder brought back feelings about it	○	○	○	○	○
I had trouble staying asleep	○	○	○	○	○
Other things kept making me think about it	○	○	○	○	○
I felt irritable and angry	○	○	○	○	○
I avoided letting myself get upset when I thought about it or was reminded of it	○	○	○	○	○
I thought about it when I didn't mean to	○	○	○	○	○
I felt as if it hadn't happened or wasn't real	○	○	○	○	○
I stayed away from reminders of it	○	○	○	○	○
Pictures about it popped into my mind	○	○	○	○	○
I was jumpy and easily startled	○	○	○	○	○
I tried not to think about it	○	○	○	○	○
I was aware that I still had a lot of feelings about it, but I didn't deal with them	○	○	○	○	○
My feelings about it were kind of numb	○	○	○	○	○
I found myself acting or feeling like I was back at that time	○	○	○	○	○
I had trouble falling asleep	○	○	○	○	○
I had waves of strong feelings about it	○	○	○	○	○
I tried to remove it from my memory	○	○	○	○	○
I had trouble concentrating	○	○	○	○	○
Reminders of it caused me to have physical reactions, such as sweating, trouble breathing, nausea, or a pounding heart	○	○	○	○	○
I had dreams about it	○	○	○	○	○
I felt watchful and on-guard	○	○	○	○	○
I tried not to talk about it	○	○	○	○	○

Confidential

Please indicate your agreement with the following statements during the COVID-19 pandemic.

	Never	Rarely	Sometimes	Frequently	Always
I feel emotionally drained from my work	◯	◯	◯	◯	◯
I feel used up at the end of the workday	◯	◯	◯	◯	◯
I feel fatigued when I get up in the morning and have to face another day on the job	◯	◯	◯	◯	◯
Working with people all day is really a strain for me	◯	◯	◯	◯	◯
I feel burned out from my work	◯	◯	◯	◯	◯
I feel frustrated by my job	◯	◯	◯	◯	◯
I feel I'm working too hard on my job	◯	◯	◯	◯	◯
Working with people directly puts too much stress on me	◯	◯	◯	◯	◯
I feel like I'm at the end of my rope	◯	◯	◯	◯	◯

	Never	Rarely	Sometimes	Frequently	Always
I can easily understand how my patients/recipients feel about things	◯	◯	◯	◯	◯
I deal very effectively with the problems of my patients/recipients	◯	◯	◯	◯	◯
I feel I'm positively influencing other people's lives through my work	◯	◯	◯	◯	◯
I feel very energetic	◯	◯	◯	◯	◯
I can easily create a relaxed atmosphere with my patients/recipients	◯	◯	◯	◯	◯
I feel exhilarated after working so closely with my patients/recipients	◯	◯	◯	◯	◯
I have accomplished many worthwhile things in this job	◯	◯	◯	◯	◯
In my work, I deal with emotional problems very calmly	◯	◯	◯	◯	◯

	Never	Rarely	Sometimes	Frequently	Always

I feel I treat some patients/recipients as if they were impersonal "objects"	○	○	○	○	○
I've become more callous toward people since I took this job	○	○	○	○	○
I worry that this job is hardening me emotionally	○	○	○	○	○
I don't really care what happens to some patients/recipients	○	○	○	○	○
I feel patients/recipients blame me for some of their problems	○	○	○	○	○

97% Complete

Please indicate how you have been feeling during the COVID-19 pandemic?

	All of the time	Most of the time	Some of the time	A little of the time	None of the time
How often did you feel tired out for no good reason?	◯	◯	◯	◯	◯
How often did you feel nervous?	◯	◯	◯	◯	◯
How often did you feel so nervous that nothing could calm you down?	◯	◯	◯	◯	◯
How often did you feel hopeless?	◯	◯	◯	◯	◯
How often did you feel restless or fidgety?	◯	◯	◯	◯	◯
How often did you feel so restless you could not sit still?	◯	◯	◯	◯	◯
How often did you feel depressed?	◯	◯	◯	◯	◯
How often did you feel that everything was an effort?	◯	◯	◯	◯	◯
How often did you feel so sad that nothing could cheer you up	◯	◯	◯	◯	◯
How often did you feel worthless?	◯	◯	◯	◯	◯

Please feel free to provide any comments regarding the impact of the COVID-19 pandemic on your personal or professional/ work life. ______________________________

THANK YOU for completing this survey!
