# Supplementary material for: Personal, professional, and psychological impact of the COVID-19 pandemic on hospital workers: A cross-sectional survey
Source: PLoS One. 2022 Feb 15;17(2):e0263438. doi: 10.1371/journal.pone.0263438 (PMC8846533; doi:10.1371/journal.pone.0263438)
Supplement: S3 Appendix — (PDF) [file pone.0263438.s003.pdf]

**Appendix 3- Media releases and email invitations to the survey**

**LHSC Recruitment Email 1**

**Subject:** Invitation to participate in research survey about your experiences at the frontlines

*We all know, COVID-19 pandemic has been a challenging time for frontline healthcare workers. We are all experiencing a range of emotions. A local team of researchers is exploring the effects of the COVID-19 pandemic on frontline healthcare workers in Southwest Ontario hospitals in order to identify strategies to support frontline hospital staff during the current pandemic and any future public health emergencies.*

*LHSC is participating in this study and all staff and physicians are welcome to participate in sharing your experience. The survey will take approximately 15 minutes to complete. Your responses will not be linked to any information that would identify you.*

*More information about the study including the letter if information and survey link at:*

*https://redcap.lawsonresearch.ca/surveys/?s=E9KKL3CXYE*

Kimia Honarmand MD, Geeta Mehta MD, John Basmaji MD, Ian Ball MD, Carol Young-Ritchie RN, MScN

**LHSC Recruitment Email 2**

**[Title]** Research survey about your experiences during COVID-19 at the frontlines

All LHSC staff and physicians are invited to participate in a research study exploring the effects of the COVID-19 pandemic on frontline hospital staff in Southwestern Ontario. Findings from this study will be used to inform strategies to support frontline workers during the pandemic and other public health emergencies. More information about the study including the letter if information and survey link at:

https://redcap.lawsonresearch.ca/surveys/?s=E9KKL3CXYE

MEDIA RELEASE
For Immediate Release
August, 2020

# Examining how the pandemic affects Southwestern Ontario's frontline hospital workers

**LONDON, ON** – A team from Lawson Health Research Institute will examine the unique personal and professional impacts of the COVID-19 pandemic on frontline hospital workers in Southwestern Ontario. The region's frontline hospital workers are invited to participate in an online survey to share their experiences during the public health emergency. The goal is to understand impacts unique to our region in order to develop strategies that address the needs of hospital workers.

"It's crucial to understand the impact of this pandemic on frontline hospital workers who often face difficult decisions, including risking personal safety to care for patients and the community," explains Dr. Kimia Honarmand, Lawson Adjunct Scientist and Critical Care Physician at London Health Sciences Centre (LHSC). "Experiences are likely to vary across the province and country based on local circumstances. It's important we hear the unique perspectives of those in our region."

The team will recruit health care professionals from hospitals across Southwestern Ontario. Participants will be asked to complete one short survey with questions about their perceptions, causes of stress and coping strategies during the pandemic. The team hopes results can be used to identify strategies that address informational, training and support needs.

In addition to worries about personal safety, the team notes that frontline workers are faced with concerns about rapidly evolving information, patient surges and depletion of resources like ventilators and personal protective equipment (PPE). They suspect social media may play a role in compounding these fears.

"Compared with past outbreaks like SARS, today's digital age provides a wealth of on-demand information and the majority is unverified," says Dr. Honarmand. "While social media can be a place of solidarity and connection, it can also contribute to the spread of misinformation and fear."

The researchers also suspect that public health measures like physical distancing, while crucial to controlling the spread of disease, may contribute to a loss of social support systems.

"Taken together, these challenges can lead to stress, anxiety and burnout," says Carol Young-Ritchie, Executive Vice President, Chief Nursing Officer and Pandemic Incident Management Team Co-Lead at LHSC. "Hearing directly from those on the frontline can help guide hospital administrators and professional organizations in better supporting our people, both during the current pandemic and in future public health emergencies."

**Frontline hospital workers in Southwestern Ontario can learn more and access the survey at https://redcap.lawsonresearch.ca/surveys/?s=E9KKL3CXYE.**

-30-

**Lawson Health Research Institute:** Lawson Health Research Institute is one of Canada's top hospital-based research institutes, tackling the most pressing challenges in health care. As the research institute of London Health Sciences Centre and St. Joseph's Health Care London, our innovation happens where care is delivered. Lawson research teams are at the leading-edge of science with the goal of improving health and the delivery of care for patients. Working in partnership with Western University, our researchers are encouraged to pursue their curiosity, collaborate often and share their discoveries widely. Research conducted through Lawson makes a difference in the lives of patients, families and communities around the world. To learn more, visit www.lawsonresearch.ca.

**For more information, please contact:**

Robert DeLaet
Communications & External Relations
Lawson Health Research Institute
T: 519-685-8500 ext. 75664
robert.delaet@lawsonresearch.com
www.lawsonresearch.ca/news-events

**Connect with Lawson:**

Email Subject: A Survey of Impact of COVID-19 on Critical Care Staff

Body of email:

**A Survey of the Personal and Professional Impact of COVID-19 on Critical Care Health Care Practitioners and Staff**

On behalf of the CCCTG, we invite you to participate in a questionnaire study of the effects of the COVID-19 pandemic on critical care health care practitioners and essential support staff.

The purpose of this survey is to evaluate the work-related and non-work related concerns regarding the COVID-19 outbreak of those who work in Canadian ICUs. We seek input from nurses, physicians, respiratory therapists, pharmacists, clerical staff, environmental services, and any other ICU workers.

The aims of this study are to describe the effects of the COVID-19 pandemic (and preparations for it) on:

- Your work in the ICU
- Concerns about yourself and your family members
- Anxiety, stress, and your mental health
- Any strategies that have helped you cope

The survey takes approximately 15 min to complete. If you agree to participate, please click here **on a computer** as the survey will not load on a mobile phone.

If the link doesn't work, copy the link below into a computer's web browser: https://ddcrc.mshri.on.ca/NoviSurvey/n/COVID19ICUSurvey.aspx

If you are an ICU leader, please forward the survey to your entire inter-professional ICU team.

Participation is voluntary and submitting data implies your consent to participate. We encourage you to respond to all questions, but you can skip any questions and still continue with the survey. The information you provide will be kept anonymous & confidential.

Thank you for sharing your thoughts and opinions!

For the Canadian Critical Care Trials Group:

| | | | |
|---|---|---|---|
| Geeta Mehta | Peter Dodek | Jeanna Parsons-Leigh | Christopher Yarnell |
| Kimia Honarmand | Jennie Johnstone | Jessica Kayitesi | Catherine Eta-Ndu |
| Alex Kiss | Robert Maunder | | |

Questions?
Please email Geeta.mehta@sinaihealth.ca
Or call the Sinai Health Research Ethics Board at 416-586-4875
