# Supplementary material for: Personal, professional, and psychological impact of the COVID-19 pandemic on hospital workers: A cross-sectional survey
Source: PLoS One. 2022 Feb 15;17(2):e0263438. doi: 10.1371/journal.pone.0263438 (PMC8846533; doi:10.1371/journal.pone.0263438)
Supplement: S1 Table — (PDF) [file pone.0263438.s004.pdf]

Table S1. Psychological instrument scoring.

Instrument	Description of instrument	Response category scale	Scoring and interpretation
K10[a]	10-item scale to assess symptoms of psychological distress (including 2 subscores reflecting depressive and anxiety symptoms)	5-point Likert scale<br>1= None of the time<br>2= A little bit of the time<br>3= Some of the time<br>4= Most of the time<br>5=All of the time	Total score range: 10-50<br>10-19 = likely psychologically well<br>20-24 = mild distress<br>25-29 = moderate distress<br>30 or higher = severe distress
IES-R[bc]	22-item questionnaire evaluating PTSD symptoms in response to stressful life events	4-point Likert scale<br>0=Not at all<br>1=A little bit<br>2=Moderately<br>3= Quite a bit<br>4= Extremely	Total score range: 0-88<br>0-23 = no major concern for PTSD<br>24-32 = PTSD is a clinical concern<br>33 or higher = probable diagnosis of PTSD<br>37 or higher = scores high enough to 'suppress immune system's functioning' up to 10 years after an impact event

K10: Kessler Psychological Distress Scale; IES-R: Impact of Events Scale Revised; PTSD: post-traumatic stress disorder

[a] Kessler et al. 2002
[b]Weiss & Marmar 1997
[c] IES-R scoring based on Kawamura et al. 2001
