# Supplementary material for: Personal, professional, and psychological impact of the COVID-19 pandemic on hospital workers: A cross-sectional survey
Source: PLoS One. 2022 Feb 15;17(2):e0263438. doi: 10.1371/journal.pone.0263438 (PMC8846533; doi:10.1371/journal.pone.0263438)
Supplement: S2 Table — (PDF) [file pone.0263438.s005.pdf]

Table S2: Description of predictor variables associated with psychological burden.

Predictor Categories	Predictor variables*
**Professional activities**	
**Hospital setting**	Teaching vs. non-teaching hospital
**Profession**	Nursing professional vs. other hospital workers
**Frontline worker**	Reported caring for patients with suspected and/ or confirmed COVID-19 (y/n)
**Years of healthcare experience**	Years of healthcare experience (reference group: > 20 years)
**Risk due to PPE shortage or inadequate PPE training**	Affirmative response to questions about feeling increased personal risk due to PPE shortage or inadequate training in PPE use (y/n)
**Demographic characteristics**	
**Age group**	Age group (reference group: age > 50 years)
**Gender**	Male vs. female
**High-risk health condition**	Having a medical condition or taking medications that increases the risk of adverse COVID-19 outcomes (y/n)
**Relationship status**	Married/ common-law relationship vs. not
**Living with child/ children**	Reported living with one or more children (y/n)
**Knowing someone who contracted COVID-19**	Knowing someone who contracted COVID-19 (y/n)

* All predictor variables were entered into the regression model and the model was iteratively reduced to retain only variables with a p-value < 0.1 in the final model (using backward selection method for linear regressions and manual reduction for the ordinal regression).

PPE: Personal protective equipment
