# Supplementary material for: Personal, professional, and psychological impact of the COVID-19 pandemic on hospital workers: A cross-sectional survey
Source: PLoS One. 2022 Feb 15;17(2):e0263438. doi: 10.1371/journal.pone.0263438 (PMC8846533; doi:10.1371/journal.pone.0263438)
Supplement: S3 Table — (PDF) [file pone.0263438.s006.pdf]

Table S3. Psychological distress, depression, anxiety, and post-traumatic stress symptoms among hospital workers during the COVID-19 pandemic by professional role.

Domain	All respondents	Nursing	Physicians	Other hospital staff	Test statistic* (p-value)
**Total K10 score**					
Median (IQR)	13.0 (7.0-20.0) N = 923	14.0 (8.0-20.0) N=391	9.0 (5.8-14.0) N=78	13.0 (6.0-20.0) N=454	H = 13.49 (0.001)[1]
**K10 psychological distress severity, n (%)**					
Minimal	690 (74.8)	287 (73.4)	70 (89.7)	333 (73.3)	
Mild	126 (13.7)	57 (14.6)	3 (3.8)	66 (14.5)	
Moderate	63 (6.8)	30 (7.7)	3 (3.8)	30 (6.6)	
Severe	44 (4.8)	17 (4.3)	2 (2.6)	25 (5.5)	
**K10 depression subscore**					
Median (IQR)	8.0 (4.0-12.0) N=931	8.0 (4.0-12.0) N=395	5.0 (3.0-9.0) N=78	8.0 (4.0-12.0) N=458	H = 12.38 (0.002)[2]
**K10 anxiety subscore**					
Median (IQR)	5.0 (2.0-8.0) N=945	5.0 (3.0-8.0) N=398	3.0 (2.0-6.0) N=80	5.0 (2.0-8.0) N=467	H = 12.49 (0.002)[3]
**Total IES-R score**					
Median (IQR)	23.0 (11.0-36.0) N = 849	26.0 (13.0-37.0) N=363	13.0 (7.0-28.0) N=71	23.0 (10.0-36.0) N=415	H = 17.94 (<0.001)[4]
**IES-R post-traumatic stress severity, n (%)**					
PTSD not a clinical concern	426 (50.2)	166 (45.7)	51 (71.8)	209 (50.4)	
Partial PTSD	134 (15.8)	66 (18.2)	5 (7.0)	63 (15.2)	
Cut-off for probable PTSD	289 (34.0)	131 (36.1)	15 (21.1)	143 (34.5)	

Data are presented as N (%).

K10: Kessler Psychological Distress Scale; IES-R: Impact of Events Scale Revised; PTSD: post-traumatic stress disorder; IQR: interquartile range

* Independent-samples Kruskal-Wallis Test.

1 Pairwise comparisons for K10 psychological distress – Physicians vs. Nursing: H = -121.24, p < 0.001; Physicians vs. Other: H = -103.43, p = 0.002; Other vs. Nursing: H = 17.81, p = 0.33.
2 Pairwise comparisons for K10 Depression subscore – Physicians vs. Nursing: H = -116.20, p < 0.001; Physicians vs. Other: H = -104.50, p = 0.001; Other vs. Nursing: 11.702, p = 0.64
3. Pairwise comparisons for K10 Anxiety subscore – Physicians vs. Nursing: H = -117.69, p < 0.001; Physicians vs. Other: H = -96.49, p = 0.003; Other vs. Nursing: H = 21.20, p = 0.25
4. Pairwise comparisons for IES-R total score – Physicians vs. Nursing: H = -134.66, p < 0.001; Physicians vs. Other: H = -109.69, p < 0.001; Other vs. Nursing: H = 24.97, p = 0.16
