# Supplementary material for: Personal, professional, and psychological impact of the COVID-19 pandemic on hospital workers: A cross-sectional survey
Source: PLoS One. 2022 Feb 15;17(2):e0263438. doi: 10.1371/journal.pone.0263438 (PMC8846533; doi:10.1371/journal.pone.0263438)
Supplement: S4 Table — (PDF) [file pone.0263438.s007.pdf]

Table S4. Thematic content analysis of open-ended responses

Themes/ Subthemes		Illustrative Quotes
**Personal Impact**		
**Personal coping & wellness**	Maintaining a positive outlook	*"I feel blessed with strong coping skills. I enjoy helping others that don't have this ability and just lend a ear when needed. Stay positive at all times. Which is not that difficult for me."*<br><br>*"…My focus on the good is helping me manage through all of the changes…"*
	Fear of contracting COVID-19	*"I developed a lot of anxiety, and constantly would convince myself that I had it. This resulted in time of work to get tested and even more stress because I was still constantly self monitoring for symptoms, even after being tested."*
	New and exacerbated psychological symptoms	*"It has brought back severe panic attacks and I have had to restart medication because of it."*<br><br>*"I suffer from mental illness so these feelings were intensified during Covid-19"*<br><br>*"COVID-19 Pandemic impacting my mental health more than I anticipated. I am generally a happy individual but not being able to workout, play sports, see my friends on a regular basis put me in dark moods frequently".*
	Fear of the unknown	*"I often felt like the anticipation of what was to come was as tiresome or moreso than the job itself. It was exhausting and very anxiety provoking.:*<br><br>*"Now I fear for the "second wave" and the stress and uncertainty of what the future of Covid bring us."*
	Cognitive overload	*"It is more stressful working with COVID than types of infectious diseases because there is no reprieve. It is everywhere in the news, socially everything changed, and there is such a sense of heightened emotions (especially at the start of the pandemic) when working in close contact with these patients."*<br><br>*"…There is no outlet or escape from the stress of the pandemic… with Covid-19, everyone is impacted. That all-encompassing feeling has been the hardest aspect for me."*
	Feelings of isolation	*"…It's hard to relate to others who are not in the thick of the pandemic, having to keep it together and be the rock for the community when others just get to hide out at home and wait for this all to go away."*<br><br>*"Have felt very isolated. Not many people understand other than other health care workers."*<br><br>*"I have moved out of my parents house during the pandemic to stay away from my pregnant sister in law and my grandparents. This was the biggest and hardest struggle that I have faced since April mentally."*

**Impact on family life**	Struggles with work-life balance	*"The issues at my workplace because of the pandemic has negatively impacted my personal life. I find it harder to leave work behind when I come home because of how hard it has become."*<br><br>*"I felt that I had no time or space for my own needs or wellbeing because I was in survival mode both at work and in my personal life."*
	Balancing role as parent & healthcare worker	*"Childcare has been my biggest stressor, particularly when my work schedule was extremely inconsistent and unpredictable.*<br><br>*".... felt incredible pressure to be consistently available to work despite the needs of young children at home and spouse's work needs."*<br><br>*"My husband and I both were expected to work outside the home and were constantly scrambling for childcare."*<br><br>*"Worrying about how to work and provide care to my children has been my greatest struggle… we both work for [the hospital] and it has been a nightmare trying to work around each others schedule and care for our children."*<br><br>*"There were really no solutions or flexibility offered to full-time single parents during the pandemic. …"*
	Support from family/ spouse	*"My husband was home with my child during the pandemic which greatly relieved the pressures of my home life. This improved the balance of my work/life responsibilities. Had I not had this resource, I don't think I would have managed as well as I had."*
**Relationship with the community**	Others' non-adherence with public health measures	*"Having to constantly re-iterate to family and friends the importance of social distancing and then realizing that they are not doing so is the exhausting part."*<br><br>*"I feel betrayed by the public who are unable to follow rules and thus put me at risk."*
	Fear of exposing others	*"There is always the fear of transmitting COVID to others, even though proper PPE is available and used properly, if only because of paranoia."*<br><br>*"There is a CONSTANT stress/worry about what if I infect my severely asthmatic son…."*
	Perceived stigmatization due to job	*"The reaction from the community and being in contact with hospital staff "You are a hero.....but stay away from me" This attitude is very disheartening…"*<br><br>*"…I am having a hard time finding a social circle because I am a HCP and no one seems to want to be around me because they are scared…"*
**Professional Impact**		
**Work environment & activities**	Job satisfaction	*"… I've maintained hope, joy and personal satisfaction from the work that I do every day."*<br><br>*"I feel resilient in my role at the hospital and in life, but I fear for those that are less well adjusted to this new world."*

	Increased workload & responsibilities	*"Getting fatigued with the extra steps I have to take to do my work because of the pandemic."*<br><br>*"… patient ratios are dangerous especially on medicine units (regularly 6 patients) which makes it difficult for me to really enjoy my job and the interactions I have with patients."*<br><br>*"We all work so hard and the work never ends. Taking time off only creates more work for our return because no one is there to fill in during our absence."*
	Modified work duties (including redeployment)	*"…Bill 195 has also been stressful since we are constantly redeployed to various floors and have no say. It is dangerous not only to us but to the patients since we don't have training on those floors and have to navigate ourselves and do our job as safely as we can which is a very hard thing to do when the floors generally are busy and high demand…"*<br><br>*"I was re-deployed without much conversation from my manager (regarding where I would go/my skill set - little notice (less than 48 hrs) regarding my redeployment and return to clinic - very little job training for pandemic…"*
	Staff shortages	*"Staffing not able to give you time off due to staff shortages."*<br><br>*"It's hard to get staff to come in, we are exhausted. Short staffed all the time..."*
	Lack of flexibility in work arrangements	*"It is disappointing that our workplace does not support us working from home, even if the type of work we do would allow for this…"*<br><br>*"It would be helpful if flexibility were given during this time to those who could work from home, especially those with young kids.f"*
**Concerns about patient care/ wellbeing**	Uncertainty about adequacy of patient care	*"I feel I cant provide high quality care as much as i would like to during this stressful time and it is morally and ethically exhausting."*<br><br>*"Feelings of burnout related to new expectations/ changing approach to care, not getting adequate direction on how to provide this care and not feeling that I am able to adequately provide the care that is needed."*
	Distress due to restricted hospital visitation policies	*"I feel that the hardest is seeing the impact of social isolation on patients - it affects them socially, emotionally and physically - and it is frustrating for me to see them lose abilities due to the limited activity they do and how it impacts their daily lives that lack of interaction with others and with the isolation…"*<br><br>*"Moral distress when leadership had Spiritual Care stay off the unit unless absolutely necessary and I found that there was a lot of absolutely necessary patient distress. Patients seemed isolated and abandon at time without anyone seeing them including nurses staying out of rooms out as much as possible."*

	Concerns about delayed care for patients with other illnesses	*“Things have normalized now, but it is increasingly busy as we see patients that have gotten deconditioned with the lock-down.”*<br><br>*“More and more people are presenting to the first time with advanced cancer with complications because they are afraid to come to the hospital. This needs to be remedied.”*<br><br>*“The fear has taken over, and the risks of COVID are far less than the risks of all these untreated conditions and especially the number of patients who are now going to wait forever for important surgeries.”*
**Relationship with colleagues**	Colleagues as a support network	*“…The support of my colleagues was the most help to get me through.”*<br><br>*“Having such great coworkers and family have been a great help…”*<br><br>*“…[I] appreciate the support of colleagues”*
	Resentment towards colleagues due to differences in pandemic pay policies	*“The difference in pandemic pay and compensation had a HUGE impact on me and my feelings towards those on the team who received the pay (and at times were not grateful for it)…”*<br><br>*“Pandemic pay – was a further divide in healthcare – to place an “us vs. them” in my shared office …*
	Concerns about personal exposure to COVID-19 from colleagues	*“Sometimes feel paranoid that my colleagues would “drop the ball” but not strictly adhering to infection control practices.”*<br><br>*“I am frustrated that my co-workers often do not follow guidelines forcing me to be extra vigilant at the workplace to protect myself and my family.”*
**Relationship with hospital leadership/ management**	Delays in implementation of protective measures	*“Everything felt two weeks behind what it should have been – watching the world and the steps they were taking.”*<br><br>*“I felt much stress at the beginning of Covid when the hospital was slow to implement restrictions.”*
	Exacerbated stress due to hospital policy changes [related & unrelated to the pandemic]	*“the beginning of covid was very stressful as things changed daily with protocols leaving you to think am I protected…”*<br><br>*“Management also felt this was a good time to make changes that were not covid related and it added to the stress of coming to work.”*
	Feeling unheard/ limited consultation with frontline workers regarding policies	*“Frontline staff have not been consulted or been able to contribute how to implement these changes in a less stressful fashion. I do not feel that I have had a voice at all, or that any suggestions I have made with regard to patient or staff safety have been given any credence.”*<br><br>*“…No advice was sought from the clinicians/staff as to what their recommendations were for handling the situation and outpatient care seemed to be put on the backburner which was difficult for the clinical caregivers.”*

	Feeling undervalued/ underappreciated	*"… The sense of teamwork has significantly diminished, we don't feel we have adequate leadership or that leadership has our best interests in mind, and I certainly don't feel appreciated."*<br><br>*"COVID has altered my practice and day to day significantly, and I find it challenging to cope with other changes to my workday with little recognizance of this strain by management."*<br><br>*"…It is very clear to me that even my own manager does not value or understand the work that I do."*
**Personal protective equipment**	Shortages in supply & limitations in access	*"I feel very angry with my employers as I feel that they did not do their best to ensure that we were correctly fitted for the n95 masks."*<br><br>*"When the shortage of PPE started, it was very difficult not to feel like we were expendable."*
